# Metabolic systems approaches update molecular insights of clinical phenotypes and cardiovascular risk in patients with homozygous familial hypercholesterolemia

**DOI:** 10.1186/s12916-023-02967-8

**Published:** 2023-07-27

**Authors:** Zhiyong Du, Fan Li, Long Jiang, Linyi Li, Yunhui Du, Huahui Yu, Yan Luo, Yu Wang, Haili Sun, Chaowei Hu, Jianping Li, Ya Yang, Xiaolu Jiao, Luya Wang, Yanwen Qin

**Affiliations:** 1 Key Laboratory of Remodeling-Related Cardiovascular Diseases, Ministry of Education, National Clinical Research Center for Cardiovascular Diseases, Beijing Anzhen Hospital, Capital Medical University, Beijing, 100029 China; 2 Beijing Institute of Heart Lung and Blood Vessel Disease, Beijing, 100029 China; 3 Department of Cardiology, The Second Affiliated Hospital of Nanchang University, Nanchang, 330006 Jiangxi Province China; 4 Department of Cardiology, Peking University First Hospital, Beijing, 100034 China; 5 Suzhou Municipal Hospital, Suzhou, 215002 Jiangsu Province China; 6 Key Laboratory of Cardiovascular Intervention and Regenerative Medicine of Zhejiang Province, Department of Cardiology, College of Medicine, Sir Run Run Shaw Hospital, Zhejiang University, Hangzhou, 310020 Zhejiang Province China

**Keywords:** Homozygous familial hypercholesterolemia, Metabolomics, Inflammation, Corneal arcus, Xanthomas, Aortic stenosis, Atherosclerotic cardiovascular disease

## Abstract

**Background:**

Homozygous familial hypercholesterolemia (HoFH) is an orphan metabolic disease characterized by extremely elevated low-density lipoprotein cholesterol (LDL-C), xanthomas, aortic stenosis, and premature atherosclerotic cardiovascular disease (ASCVD). In addition to LDL-C, studies in experimental models and small clinical populations have suggested that other types of metabolic molecules might also be risk factors responsible for cardiovascular complications in HoFH, but definitive evidence from large-scale human studies is still lacking. Herein, we aimed to comprehensively characterize the metabolic features and risk factors of human HoFH by using metabolic systems strategies.

**Methods:**

Two independent multi-center cohorts with a total of 868 individuals were included in the cross-sectional study. First, comprehensive serum metabolome/lipidome-wide analyses were employed to identify the metabolomic patterns for differentiating HoFH patients (*n* = 184) from heterozygous FH (HeFH, *n* = 376) and non-FH (*n* = 100) subjects in the discovery cohort. Then, the metabolomic patterns were verified in the validation cohort with 48 HoFH patients, 110 HeFH patients, and 50 non-FH individuals. Subsequently, correlation/regression analyses were performed to investigate the associations of clinical/metabolic alterations with typical phenotypes of HoFH. In the prospective study, a total of 84 HoFH patients with available follow-up were enrolled from the discovery cohort. Targeted metabolomics, deep proteomics, and random forest approaches were performed to investigate the ASCVD-associated biomarkers in HoFH patients.

**Results:**

Beyond LDL-C, various bioactive metabolites in multiple pathways were discovered and validated for differentiating HoFH from HoFH and non-FH. Our results demonstrated that the inflammation and oxidative stress-related metabolites in the pathways of arachidonic acid and lipoprotein(a) metabolism were independently associated with the prevalence of corneal arcus, xanthomas, and supravalvular/valvular aortic stenosis in HoFH patients. Our results also identified a small marker panel consisting of high-density lipoprotein cholesterol, lipoprotein(a), apolipoprotein A1, and eight proinflammatory and proatherogenic metabolites in the pathways of arachidonic acid, phospholipid, carnitine, and sphingolipid metabolism that exhibited significant performances on predicting first ASCVD events in HoFH patients.

**Conclusions:**

Our findings demonstrate that human HoFH is associated with a variety of metabolic abnormalities and is more complex than previously known. Furthermore, this study provides additional metabolic alterations that hold promise as residual risk factors in HoFH population.

**Supplementary Information:**

The online version contains supplementary material available at 10.1186/s12916-023-02967-8.

## Background

Familial hypercholesterolemia (FH) is an inborn errors of metabolism disorder caused by mutations in the gene-encoding low-density lipoprotein receptor (LDLR). Rare mutations in apolipoprotein B (APOB) and proprotein convertase subtilisin/kexin type 9 (PCSK9) genes may also produce the FH phenotype [1, 2]. Heterozygous FH (HeFH) carrying single mutation is relatively common and globally occurs in one of every 200–300 people [3]. Homozygous FH (HoFH) is an umbrella term that encompasses a spectrum of genetic diagnoses. Patients with HoFH have two pathogenic variants either in the same or two different mutations in the causative genes. Worldwide, HoFH is an extremely rare and life-threatening condition with a prevalence between 1:160,000 and 1:400,000 [4–6]. In China, it was estimated that there are approximately 5000 HoFH individuals, in line with a prevalence of 1:600,000 [7, 8]. Due to the extremely rare prevalence, the metabolic profiles of human HoFH are largely unknown.

Extreme elevation of circulating low-density lipoprotein cholesterol (LDL-C) is the critical discriminator for clinical diagnosis of HoFH [6]. Compared to HeFH population, HoFH is characterized by a more aggressive phenotype of circulating LDL-C levels and cardiovascular consequences [9, 10]. HoFH patients can develop atherosclerotic cardiovascular disease (ASCVD), supravalvular aortic stenosis, or cardiac death at very young ages [10, 11]. The widespread acceptance is that the major cause for these adverse cardiovascular complications is extremely increased LDL-C [12]. To date, lipoprotein apheresis and liver transplantation remain the most effective but expensive lipid-lowering therapy (LLT) options for treating HoFH [10–14].

In addition to LDL-C, evidence for other potential cardiovascular risk factors in patients with FH is also emerging. Interestingly, several observations and experiments in the HeFH population failed to find associations between LDL-C and ASCVD events and indicated that lipoprotein (a) [Lp(a)] and several inflammatory cytokines might be strongly driving factors for cardiovascular risk in the HeFH population [15–17]. Although advanced LLT measures for decreasing LDL-C have significantly improved the outcomes of supravalvular aortic stenosis in HoFH patients, recent evidence has shown that LLT cannot alter calcific aortic stenosis progression once present, even with lipoprotein apheresis, liver transplant, or normalization of LDL-C levels [18]. Our recent study revealed that adolescent patients developed myocardial infarction 1 year after liver transplantation, even with a lower LDL-C level < 2.5 mmol/L [8]. These evidences suggest that the cardiovascular risk driving factors in HoFH are complicated and have not been fully understood.

Metabolomics and lipidomics are amenable metabolic systems approaches opening a window to mechanistically interpret inborn errors of metabolism disorder [19, 20]. Deep metabolic profiling can provide a holistic view of small-molecule metabolite profiles controlled at the level of the genome and predict clinical consequences [21]. Importantly, numerous studies highlight that multiple metabolite markers can contribute to the development of a variety of cardiovascular diseases [22, 23]. Recent metabolomic studies showed that HeFH patients and genetic animal models that mimic human HoFH were characterized by profound metabolite alterations implicated in the progression and development of atherosclerosis [24–26]. Furthermore, our recent study of 142 HoFH patients indicated that different LDLR genotypes could lead to significant heterogeneity in the serum metabolome phenotype of patients with HoFH [27]. These studies demonstrated that the metabolic characteristics in HoFH might not be fully explained by LDL-C.

**Fig. 1:**
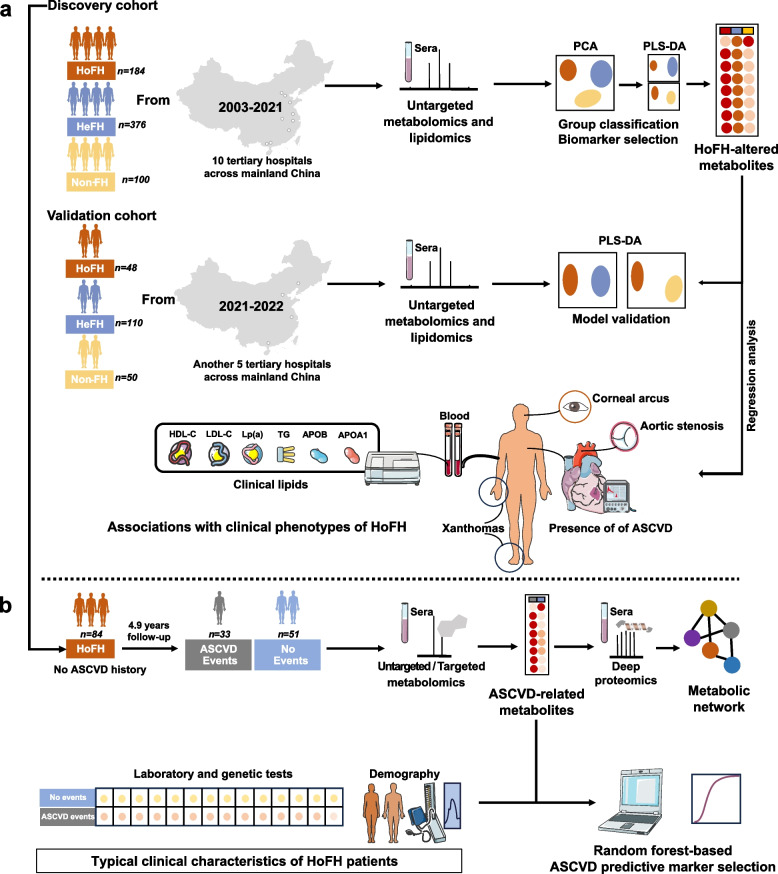
Study overview. **a** Experimental procedure of cross-sectional study. Two independent multi-center cohorts were included in the global metabolomic/lipidomic analyses. Multivariate statistical analysis was used to discover and validate the specific metabolite patterns of HoFH. Regression analysis was employed to investigate the associations of HoFH-related metabolites with typical clinical phenotypes of HoFH patients. **b** Experimental procedure of follow-up study; 84 HoFH patients with available follow-up information were enrolled from the discovery cohort. Targeted metabolomics and deep proteomics were performed to investigate the ASCVD-associated metabolic network at multi-omic levels. Random forest algorithm was used to select the important metabolic/clinical markers in predicting ASCVD events

Here, we sought to systematically characterize the clinical phenotypes, clinical laboratory lipid profiles, serum metabolome, and lipidome profiles of the largest panel of HoFH patients collected from two independent multi-center HoFH cohorts across mainland China. We also aimed to investigate the associations of key metabolic/clinical alterations with aggressive phenotypes and cardiovascular consequences in HoFH patients (Fig. 1).

## Methods

### Subjects and study design

In the cross-sectional study (Fig. 1a), two independent multi-center cohorts with a total of 868 individuals were included in the global metabolomic/lipidomic analyses. In the discovery cohort, 184 HoFH patients, 376 HeFH patients, and 100 non-FH subjects were enrolled from the Familial Hypercholesterolemia Families Cohort (FHFC) affiliated to Beijing Anzhen Hospital between 2003 and 2021 [8, 28]. FHFC is an ongoing and prospective multi-center cohort study (ten tertiary hospitals) designed to investigate the genes, imaging, treatment, and prognostic factors of FH patients across mainland China. A total of 48 HoFH patients, 110 HeFH patients, and 50 non-FH individuals recruited at another independent multi-center FH cohort (five third-class hospitals) affiliated to Peking University First Hospital constituted the external validation phase between January 2021 and January 2022 [27]. Verbal and written consent was obtained from all subjects. This study complies with the Declaration of Helsinki and was approved by the Ethics Committee of Beijing Anzhen Hospital of the Capital University of Medical Sciences and the Ethics Committee of Peking University First Hospital. This study has been registered with www.chictr.org.cn/index.aspx (number: ChiCTR1900022156).

In the prospective study (Fig. 1b), a total of 84 HoFH patients without a history of ASCVD events and liver transplant surgery were enrolled from the discovery cohort. Patients in the validation cohort were not included, due to their limited follow-up time. Untargeted/targeted metabolite profiling and deep proteomics were performed to investigate the ASCVD-related metabolic alterations. During the follow-up, all 84 HoFH patients received continuous lipid-lowering therapy and strict diet control. Fatal and non-fatal ASCVD events were recorded. Fatal events included cardiac deaths, non-fatal events included non-fatal myocardial infarction, unstable or stable angina requiring revascularization, or non-fatal ischemic stroke.

### Diagnosis of familial hypercholesterolaemia

FH diagnosis was established by means of the diagnostic criteria of the Dutch Lipid Clinic Network (DLCN) and HoFH International Clinical Collaborators (HICC) registry [5, 7, 8], and only patients with a DLCN score ≥ 6 (main criteria: untreated LDL-C ≥ 4.7 mmol/L; either xanthomas or corneal arcus; FH or premature ASCVD in first-degree relatives; genetically confirmed FH) were enrolled. All HoFH subjects in this study were genetically confirmed to have two mutant alleles at the genes encoding LDLR, APOB, proprotein convertase subtilisin/kexin type 9 (PCSK9), or LDLR adaptor protein 1. Genotyping was obtained from patients if it had been performed or tested according to our previously reported methods [8, 29]. Genetically diagnostic HoFH individuals can be either true homozygotes, compound heterozygotes, or double heterozygotes. LDLR functionality was annotated as previously described and categorized as either null (< 2%) or defective (2% to 25%) [6, 8, 27, 30]. LDLR status was classified as at least one null mutation (null/null or null/defective) or not (defective/defective). Non-FH was defined as subjects with lower levels of untreated LDL-C than 4.7 mmol/L.

### Sample collection and clinical laboratory lipid profiles

Blood samples were collected from the antecubital vein of patients who meet the requirements of an overnight fasting state of 10–12 h. Serum was separated by centrifugation at 3000 × *g* for 20 min and then stored at − 80 °C until analysis. The serum levels of LDL-C, total cholesterol (TC), high density lipoprotein cholesterol (HDL-C), Lp(a), APOB, apolipoprotein A1 (APOA1), and triglycerides (TG) were determined using an automatic biochemistry analyzer (Beckman AU 5400, Brea, USA). The LDL-C-year score was calculated as previously described: LDL-C max × (age at diagnosis/initiation of statin) + LDL-C at inclusion × (age at inclusion − age at diagnosis/initiation of statin therapy) [31].

### High-throughput metabolomics and lipidomics

The hydrophilic and lipophilic metabolites were extracted from serum by using liquid–liquid extraction as follows: 150 μL serum was extracted by fourfold volume of ice-cold chloroform: methanol (2:1, v/v) containing of internal standard mixtures of L-arginine-*d*_7_, L-phenyl-*d*_5_-alanine, L-leucine-5, 5, 5-*d*_3_, stearic acid-18, 18, 18-*d*_3_, arachidonic acid-*d*_5_, cholic acid-2, 2, 4, 4-*d*_4_, lysophosphocholine (19:0)-*d*_5_, ( ±)15-hydroxyeicosatetraenoic acid-*d*_8_, prostaglandin E_2_-*d*_4_, phosphocholine (18:0/20:4)-*d*_11_, L-carnitine-*d*_3_, stearoyl-L-carnitine-*d*_3_, ceramide (d18:1 /16:0)-*d*_7_, cholesterol-*d*_7_, and palmitoyl-*d*_9_ lysophosphatidic acid. The mixture was vortexed and centrifuged at 14,500 rpm for 10 min at 4 °C. Then the upper aqueous phase (hydrophilic metabolites) and the lower organic phase (lipids) were separately collected for metabolomics and lipidomics, respectively. All the supernatant was transferred into a clean dry tube and evaporated to dryness. The dried residue was stored at − 80 °C. Following the above protocol, a quality control (QC) sample was also prepared by mixing equal aliquots from each sample. Metabolomic and lipidomic analyses were performed on UHPLC-Q-Exactive HF MS (Thermo Fisher Scientific, Waltham, MA, USA) as previously described [32, 33]. Detailed methods are depicted in Additional file 1.

### Targeted metabolomics and deep proteomics

The validation of the identified ASCVD-associated metabolites from untargeted profiling was performed by Metware Ltd. (http://www.metware.cn/) using multiple reaction monitoring (MRM) model based on the AB Sciex QTRAP 6500 LC–MS/MS platform and chemical standards. Protein corona preparation and data-independent acquisition-based deep proteomics analysis were performed by Novogene Co., Ltd. (https://www.novogene.com/) based on the Thermo Fisher Q Exactive TM HF-X LC–MS/MS platform [34]. Detailed methods are provided in Additional file 1.

### Statistical analysis

For clinical variables, continuous data are presented as the mean and standard deviation (means ± SD), and the data not normally distributed are expressed as medians and interquartile ranges [IQR]. One-way ANOVA and consequent Scheffé tests were used for the comparison of three groups. The unpaired two-tailed Student’s *t* test and Mann Whitney *U* test were used for the two-group comparison of normally distributed data and non-normally distributed data, respectively. Categorical variables were summarized by frequency (*N*) and percentages (%) and compared using chi-square test. *P* < 0.05 was considered significant. Cox regression models with hazard ratios (HRs) and 95% confidence intervals (CIs) were performed using SPSS Statistics 26. Forest plot was performed to display the HRs and 95% CIs of association for clinical variables with the occurrence of ASCVD events. All analyses were performed by using SPSS Statistics 26 (IBM Corp, New York, USA).

The semi-quantitative/quantitative values of metabolites obtained from untargeted metabolic analysis were calculated by using the isotope-labeled internal standards. The normalized data matrix of metabolites was log-transformed and auto-scaled to maintain a symmetrical and comparable distribution. Unsupervised principal component analysis (PCA) was applied to gain a comprehensive view of the sample distribution of HoFH, HeFH, and non-FH groups and assess the outlier samples in both cohorts. The optimized number of principal components (PCs) was determined by Q2 and R2 values as follows: when the cumulative Q2 values of the first *n* PCs were decreased after adding a new PC, the first *n* PCs were selected as the optimized PCs for constructing PCA models. A cumulative Q2 value > 0.5 and cumulative *R*2 value > 0.5 indicate a good predictivity and explanatory ability in the established PCA model.

Supervised partial least square discriminant analysis (PLS-DA) was performed to use the group label to maximize the group separation and screen of the HoFH-altered metabolic variables in the discovery cohort. The optimal number of latent variables (components) needed to build the PLS-DA model was determined by three performance measures of leave-one-out cross validation (LOOCV), including the sum of squares captured by the model (R2), the cross-validated R2 (Q2), and the prediction accuracy (accuracy). The optimal number yielding the minimum classification error was selected as follows: when the cumulative Q2 values of the first *n* components were reduced after adding a new component, *n* was selected as the optimal number of the components for constructing the PLS-DA model. A cumulative Q2 value > 0.5, cumulative *R*2 value > 0.5, and accuracy > 0.8 were considered reliable for the establishing PLS-DA model. The permutation test and analysis of variance testing of cross-validated predictive residuals (CV-ANOVA) *P* value were used to assess the reliability of the established PLS-DA model. The most important metabolites that were significantly correlated with the group labels/classification were selected by using the variable importance projection (VIP) and modeled covariation p-scaled correlation coefficient [p(corr)] plots of PLS-DA model. The modeled covariation *P* value expresses the importance of the variables in the selected component. A high absolute p(corr) means a very high reliability (modeled correlation) while the square has a high model influence. The VIP value > 1.0 and absolute p(corr) value > 0.1 represent a significant importance of the metabolic variables in differentiating HoFH from HeFH or non-FH. The statistical significance of the selected metabolic variables from the PLS-DA model was further verified by univariate statistical analysis, including Student’s *t* test and Mann Whitney *U* test. A false discovery rate (FDR)-adjusted *P* value < 0.05 was considered significant.

Based on the differentiated metabolites identified in the discovery cohort, PLS-DA was also performed to test whether these altered metabolites could accurately differentiate HoFH patients from non-FH and HeFH individuals in the validation cohort. The optimal number of PLS-DA components was determined by LOOCV plot. The reliability of the established PLS-DA models was evaluated by Q2 values, the number of misclassifications in posterior classification probability plot (100 cross-validation), and the *P* values of permutation test (*n* = 1000 times). All multivariate statistical analysis (MVA) were performed with SIMCA-P software (v14.0, Umetrics, Umea, Sweden) and MetaboAnalyst (http://www.metaboanalyst.ca/).

Random forest (RF), an ensemble supervised learning method for variable reduction and selection, allows reducing variance in decision trees and has been shown to handle challenges arising from small sample sizes and feature numbers [35]. In this study, RF was applied to estimate the association between metabolic/clinical variables and ASCVD events and select the important variables using R. software (https://cran.r-project.org/index.html). The dataset was divided into two-thirds for training and one-third for testing. The function Random Over-Sampling Examples (ROSE) from the R package “ROSE package” was applied to reduce the data imbalance rate. The *ntree* value was set at 500, the *mtry* values were set at 5, and the other hyperparameters were set at default settings of R package “Random Forest”. The variable importance was evaluated by using the values of mean decrease accuracy. Monte Carlo cross validation (MCCV) was used to generate multivariate RF model for selecting the optimal biomarker panel. In each MCCV, two thirds (2/3) of the samples are used to evaluate the performance of different RF models using different numbers of top important metabolic/clinical variables. Then, the left 1/3 of the samples were used to validate the performances of the established models. The performance of different multivariate RF models was assessed using the values of area under the receiver-operating characteristic curve (AUC-ROC) and predictive accuracy. Kaplan–Meier estimation with the log-rank test was performed to determine the overall ASCVD event-free survival time according to the concentrations of the selected metabolites.

Volcano plots (log_2_ fold change vs. -Log_10_ *P* value), treemap, and chord diagram were generated by using bioinformatics platform (http://www.bioinformatics.com.cn). Heatmap analyses were conducted using TBtools software [36]. Spearman’s rank coefficients were used to investigate the correlation between metabolites and clinical lipids. Pearson correlation analysis was used to assess the correlation between quantitative and semi-quantitative levels of metabolites. Regression plots were performed to explore the correlations between clinical/metabolic variables and corneal arcus/xanthomas. The score plots of PCA and orthogonal PLS-DA models were applied to explore the associations between metabolites and LLT/CVD history. All analyses were performed on SPSS Statistics 26, SIMCA-P software, and bioinformatics platform.

### Pathway analysis

Pathway analysis was performed utilizing Quantitative Enrichment Analysis in MetaboAnalyst based on the Small Molecule Pathway Database (SMPD) and Kyoto Encyclopedia of Genes and Genomes (KEGG) pathway databases. The pathway enrichment ratio and *P* value were calculated based on the log-transformed quantitative matrix of the differentiated metabolites obtained from the comparisons of HoFH, HeFH, and non-FH individuals. A high enrichment ratio value indicated an important impact of the input metabolites in the selected pathway. FDR-adjusted *P* value < 0.05 was considered as significant. The latent relationship network between functional pathways/diseases and metabolites was generated based on Function Analysis, Connect Analysis, and Path Explorer by using Ingenuity Pathway Analysis (IPA, QIAGEN Inc., German). All functional GO analyses and pathway enrichment for the differentially expressed proteins were performed on ClueGO within Cytoscape software 3.4. GO terms were categorized in biological processes, molecular functions, and cellular components. Pathway analysis was categorized in the KEGG and REACTOME pathways. The pathway enrichment and metabolite–protein interaction network of differentially expressed proteins and metabolites was performed utilizing MetaboAnalyst.

## Results

### Demographic and clinical characteristics of all participants

A total of 660 individuals participated in the discovery cross-sectional study (Fig. 1a), including 184 genetically confirmed HoFH patients (male, 50.1%), 376 HeFH patients (male, 49.2%), and 100 non-FH individuals (male, 53.0%). The clinical characteristics of the study individuals are presented in Table 1. Patients with HoFH had higher levels of LDL-C, TC, non-HDL, Lp(a), and APOB and lower levels of HDL and APOA1 than HeFH and non-FH individuals. In addition, HoFH patients had a higher prevalence ratio of corneal arcus, xanthomas, and aortic stenosis than HeFH and non-FH subjects. A total of 48 HoFH patients (male, 43.8%), 110 HeFH patients (male, 46.4%), and 50 non-FH subjects (male, 52%) from another independent multi-center institution constituted the validation cohort (Fig. 1a). As shown in Additional file 2: Table S1, the alterations in the clinical lipid profiles and the prevalences of HoFH-associated clinical complications between different groups in the validation cohort were similar to those in the discovery cohort. The detailed LLT information on both cohorts is summarized in Additional file 2: Table S2. Most of patients with HoFH had received at least two LDL-C-lowering medications.

**Table 1:** Demographic and clinical characteristics of all subjects in the discovery cohort

	HoFH (*n* = 184)	HeFH (*n* = 376)	non-FH (*n* = 100)	*P* value
Ages	20.7 ± 14.3	22.7 ± 11.2	20.0 ± 11.9	0.34
Male sex, *n* (%)	93 (50.1%)	185 (49.2%)	53 (53%)	0.79
Hypertension, *n* (%)	6 (3.3%)	11 (2.9%)	2 (2%)	0.83
Diabetes mellitus, *n* (%)	1 (0.5%)	4 (1.1%)	1 (1%)	0.83
Current smokers, *n* (%)	1 (0.5%)	9 (2.4%)	2 (2%)	0.30
ASCVD history, *n* (%)	25 (13.6%)	11 (2.9%)^a^	0 (0.0%)^b^	< 0.0001
Current corneal arcus, *n* (%)	80 (43.5%)	0 (0.0%)^a^	0 (0.0%)^b^	< 0.0001
Current xanthomas, *n* (%)	89 (48.4%)	9 (2.4%)^a^	0 (0.0%)^b^	< 0.0001
SVAS, *n* (%)	40 (21.7%)	0 (0.0%)^a^	0 (0.0%)^b^	< 0.0001
Calcific VAS, *n* (%)	38 (20.7%)	0 (0.0%)^a^	0 (0.0%)^b^	< 0.0001
LLT, *n* (%)	165 (89.7%)	264 (70.2%)^a^	37 (37%)^b^	< 0.0001
LDL-C, mmol/L	13.41 ± 5.10	4.94 ± 1.52^a^	2.15 ± 0.32^b^	< 0.0001
TC, mmol/L	15.95 ± 5.21	6.89 ± 1.67^a^	3.86 ± 0.52^b^	< 0.0001
TG, mmol/L	0.98 [0.71, 1.38]	1.27 [0.83, 1.86]^a^	0.72 [0.56, 0.89]^b^	< 0.0001
HDL-C, mmol/L	0.93 ± 0.32	1.41 ± 0.48^a^	1.49 ± 0.24^b^	< 0.0001
Non-HDL, mmol/L	15.02 ± 5.28	5.43 ± 1.81^a^	2.49 ± 0.31^b^	< 0.0001
APOB, g/L	2.55 ± 0.68	1.49 ± 0.44^a^	0.98 ± 0.27^b^	< 0.0001
APOA1, g/L	0.82 ± 0.27	1.24 ± 0.25^a^	1.32 ± 0.34^b^	< 0.0001
LP(a), mg/dL	41.0 [21.3, 73.8]	17.3 [6.0, 20.4]^a^	8.9 [5.3, 17.6]^b^	< 0.0001

Continuous data are presented as mean ± standard deviation or median [interquartile range], and categorical variables are presented as %. ANOVA and consequent post hoc test were used for continuous data. The chi-square test was used for categorical data

*CVD* cardiovascular disease, *LDL-C* low-density lipoprotein cholesterol, *TC* total cholesterol, *TG* triglycerides, *HDL-C* high-density lipoprotein cholesterol, *APOB* apolipoprotein B, *APOA1* apolipoprotein A1, *Lp(a)* lipoprotein (a), *LLT* lipid-lowering therapy, *SVAS* supravalvular aortic stenosis, *VAS* valvular aortic stenosis, *HoFH* homozygous familial hypercholesterolemia, *HeFH* heterozygous familial hypercholesterolemia, *Non-FH* non-familial hypercholesterolemia

^a^*P* value < 0.05 in the comparison of HeFH and HoFH

^b^*P* value < 0.05 in the comparison of non-FH and HoFH

### HoFH, HeFH, and non-FH subjects show distinct serum metabolite profiles

**Fig. 2:**
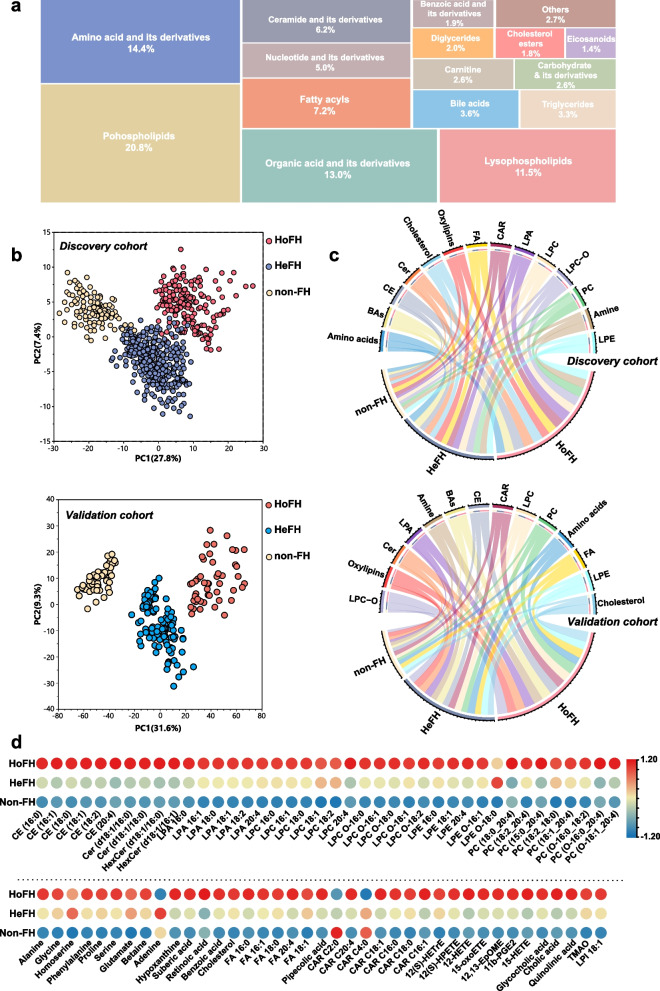
Pattern analysis of the dataset from the serum metabolome and lipidome profiling. **a** Treemap overview for the metabolic category distribution of annotated metabolites based on untargeted metabolomic/lipidomic analyses. **b** PCA score plot of the metabolic profiling data from serum samples of HoFH patients, HeFH patients, and non-FH individuals in the discovery and validation cohorts; each point represents an individual serum sample. **c** Chord diagram of the major differentiated metabolic classes across the HoFH, HeFH, and non-FH groups from the discovery and validation cohorts; the width of the curves indicates the mean percentage of each metabolic class between the study groups. Each color represents a unique metabolite class. **d** Heatmap of the differentiated metabolites that distinguished HoFH patients from HeFH and non-FH individuals in discovery cohort. HoFH homozygous familial hypercholesterolemia, HeFH heterozygous familial hypercholesterolemia, non-FH non-familial hypercholesterolemia, FA fatty acids, CE cholesterol esters, Cer ceramide, CAR acyl carnitines, LPA lysophosphatidic acid, LPI lysophosphatidylinositol, PC diacylglycerophosphocholines, LPC lyso PC, LPC-O alkyl-LPC, LPE lysophosphoethanolamines, LPE-O alkyl-LPE

After data preprocessing, a total of 885 circulating metabolites were identified in the untargeted metabolomic and lipidomic profiling, including 325 chemical standard-annotated metabolites. The full list of the annotated metabolites is listed in Additional file 3. These metabolites mainly consisted of amino acid and its derivatives, organic acids, fatty acyls, bile acids, carbohydrate and its derivatives, eicosanoids, nucleotide and its derivates, carnitine, ceramide and its derivatives, cholesterol esters, diglycerides, triglycerides, phospholipids, and lysophospholipids (Fig. 2a). Furthermore, the unsupervised PCA score plot of QC samples and the relative standard derivations of the distribution for the identified metabolites in the QC samples are shown in Additional file 2: Fig. S1 a–d, and the results indicated that the proposed LC–MS approach was robust and reproducible for further analysis.

To test whether the metabolic profiling could discriminate HoFH patients, HeFH patients, and non-FH individuals, we employed PCA analysis designed to distinguish the group separation. Based on the cumulative Q2 and *R*2 values, PCA models of the discovery and validation cohorts were established based on eight PCs and nine PCs, respectively. The two established PCA models were highlighted with satisfactory values of cumulative R2 and Q2 (discovery cohort: *R*2 = 0.58, Q2 = 0.52; validation cohort: *R*2 = 0.69, Q2 = 0.61), indicating good explanatory and predictive ability (Additional file 2: Fig. S1 e, f). The first two PC-based PCA score plots are depicted in Fig. 2b. In the discovery cohort, a clear clustering trend from non-FH individuals to patients with HeFH to those with HoFH was observed. Furthermore, a similar group separation trend among HoFH, HeFH, and non-FH was also observed in the validation cohort. These results indicated that the metabolite profiles of HoFH were significantly different from those of non-FH and HeFH.

### HoFH is characterized by profound serum metabolite alterations

To maximize the group separation and identify the differentially expressed metabolites that distinguish HoFH from non-FH and HeFH in the discovery cohort, the supervised PLS-DA models were constructed. The performance measure plots of LOOCV showed that the first three components were best for establishing PLS-DA models for differentiating HoFH from non-FH and HeFH (Additional file 2: Fig. S2a). The reliability of the established PLS-DA models was confirmed by the permutations plot and highlighted with significant CV-ANOVA *P* values (all *P* values < 0.0001; Additional file 2: Fig. S2b). The first two components-based PLS-DA score plots revealed a distinct separation of the sera metabolite profiles of HoFH patients from non-FH and HeFH individuals in the discovery cohort (Additional file 2: Fig. S2c). Then, the combined VIP and p(corr) plots of PLS-DA models were constructed to identify the important metabolic variables responsible for group separation (Additional file 2: Fig. S2d). Finally, we identified a panel of 79 serum metabolites that could significantly differentiate HoFH patients from HeFH and non-FH subjects in the discovery cohort (summarized in Additional file 2: Table S3). Furthermore, the findings obtained from PLS-DA analysis were also confirmed by the univariate analysis, as shown in the volcano plots (Additional file 2: Fig. S2e).

Next, we performed PLS-DA analysis to test the discriminatory performances of the differentiated metabolites obtained from the discovery cohort in differentiating HoFH from non-FH and HeFH in the validation cohort. The optimal number of components for building PLS-DA models were selected using the LOOCV plot (Additional file 2: Fig. S3a). We found that the cumulative Q2 values and accuracies of the first two components in two PLS-DA models were greater than 0.9, and the resultant PLS-DA score plots revealed a clear separation between HoFH and non-FH/HeFH in the validation cohort (Additional file 2: Fig. S3b). Furthermore, the optimal numbers of PLS-DA components-based posterior classification probability plots demonstrated that no HoFH individuals were predicted as non-FH or HeFH individuals in the validation cohort (Additional file 2: Fig. S3c). The reliability of the established PLS-DA models was also confirmed by the permutations plot (*n* = 1000 times; *P* values < 0.000001; Additional file 2: Fig. S3d). These findings revealed that the differentiated metabolites identified in the discovery cohort could also accurately differentiate HoFH from non-FH and HeFH in the validation cohort.

The relative percentages of major metabolic categories and mean normalized levels of differentiated metabolites were plotted as chord diagrams and heatmaps (Fig. 2b, c; Additional file 2: Fig. S3e). From the resultant graphs, it was evident that the expression levels of these differentiated metabolites in HoFH were distinct from those of HeFH and non-FH subjects in both cohorts. HoFH patients had higher levels of amino acids, fatty acids (FA), eicosanoids, cholesterol esters (CE), ceramide (Cer), lysophosphatidic acid (LPA), lysophosphoethanolamine (LPE), acyl carnitine (CAR), diacylglycerophosphocholine (PC), lysophosphocholine (LPC), alkyl-LPC (LPC-O), and bile acids.

Considering that LLT and existing ASCVD are potential metabolic regulatory factors affecting the serum metabolome and lipidome, we employed unsupervised PCA and supervised orthogonal PLS-DA analysis to assess their potential effects on the identified metabolite markers that differentiated HoFH from HeFH and non-FH. As shown in Additional file 2: Fig. S4 a–d, the metabolic alterations did not show clustering of samples by LLT status or ASCVD history in both unsupervised and supervised MVA models, indicating no discriminatory metabolite features due to differences in LLT status or ASCVD history of the study groups. The associations between these discriminatory metabolites and the clinical lipids were performed by using Spearman’s rank correlation coefficients. As expected, most of the altered small-molecule lipids that distinguished HoFH from HeFH and non-FH were strongly associated with clinical lipid makers, including LDL-C, TC, APOB, APOA1, Lp(a), HDL-C, and non-HDL (Additional file 2: Fig S4 e).

### HoFH-associated metabolites are implicated in a variety of metabolic and functional pathways

**Fig. 3:**
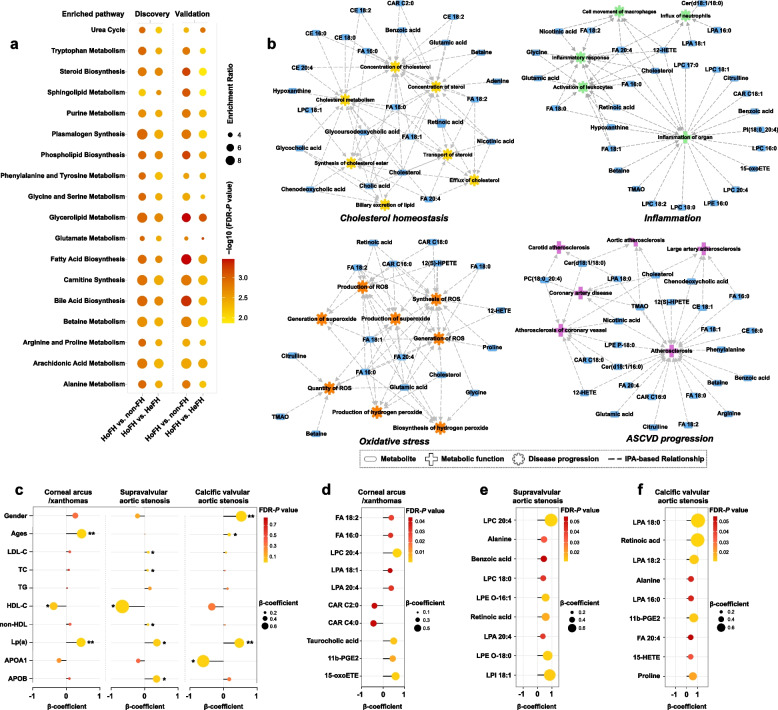
Pathway enrichment and clinical phenotype-correlation analyses of metabolic signatures. **a** Expression level-based quantitative pathway enrichment analysis of the differentiated metabolites that distinguished the HoFH group from the non-FH and HeFH groups. The bubble size refers to the enrichment ratio of the pathway and the color represents the natural log of the FDR-adjusted *P* value. **b** IPA-based functional network between the HoFH-associated metabolites and enriched biological pathways/diseases. Rectangles, nodes, and edges represent metabolites, functional pathways/diseases, and associations, respectively. **c** Correlation plot depicting the correlation of clinical lipids with corneal arcus/xanthomas, supravalvular aortic stenosis, and valvular aortic stenosis, using regression analyses (FDR-adjusted *P < 0.05; FDR-adjusted **P < 0.01). **d–f** Correlation plot depicting the relation of metabolomic features with corneal arcus/xanthomas, supravalvular aortic stenosis, or valvular aortic stenosis (FDR-adjusted *P < 0.05; FDR-adjusted **P < 0.01). LDL-C low-density lipoprotein cholesterol, HDL-C high-density lipoprotein cholesterol, Lp(a) lipoprotein (a), ApoA1 apolipoprotein A1, ApoB apolipoprotein B. Other abbreviations are shown in Fig. 1

To factually characterize the HoFH-perturbed metabolic pathways, MetaboAnalyst-based quantitative pathway enrichment analysis was performed using the quantitative matrix of the differentiated metabolites between groups. The enriched metabolic pathways and associated metabolites are summarized in Fig. 3a and Additional file 4, respectively. In the discovery cohort, the results indicated that the metabolites distinguishing HoFH patients from HeFH and non-FH individuals were significantly enriched in lipid metabolism pathways, including arachidonic acid metabolism, phospholipid metabolism, sphingolipid metabolism, and biosynthesis pathways of steroid, bile acids, carnitine, plasmalogen, and fatty acids. In addition, three hydrophilic metabolite markers (including glycine, alanine, and glutamate) were significantly enriched in the urea cycle, purine metabolism, and several amino acid metabolism pathways (Additional file 4). Furthermore, we demonstrated that the differentiated metabolites obtained from the validation cohort also showed similar influences (enrichment ratios) on the enriched pathways with high impact significance (all FDR-adjusted *P* values < 0.05; Fig. 3a). To further understand the biological function and latent diseases of HoFH-associated metabolites, a functional relationship network analysis was performed using the IPA knowledge database. The results revealed that those HoFH-altered metabolites were primarily associated with cholesterol homeostasis, oxidative stress, inflammation, and ASCVD progression (Fig. 3b). Most strikingly, the accumulation of FA species, CE species, CAR species, and lysophospholipids in the serum of HoFH patients was closely associated.

### Inflammatory and oxidative stress-related metabolites are correlated with corneal arcus and xanthomas

Corneal arcus and xanthomas are typical characteristics in HoFH individuals. Among the 232 HoFH patients in the discovery and validation cohorts, a total of 106 HoFH patients presented with corneal arcus, and all of them also presented with xanthomas. Using the merged datasets obtained from discovery and validation cohorts, we used regression analysis to assess the correlations of clinical lipids with corneal arcus/xanthomas. Although excess cholesterol (LDL-C and TC) promoting the formation of corneal arcus and xanthomas in HoFH had been well recognized [11], the results of regression analysis demonstrated that LDL-C, TC, and APOB only showed a non-statistically positive correlation with corneal arcus and xanthomas (all *P* value > 0.05; Fig. 3c). However, we found that age (β-coefficient = 0.458, *P* = 0.003), Lp(a) (β-coefficient = 0.44, *P* = 0.005), and HDL-C (β-coefficient =  − 0.376, *P* = 0.05) were positively associated with the prevalence of the corneal arcus and xanthomas.

Next, we performed univariate regression analyses to test whether HoFH-associated serum metabolites were associated with xanthomas and corneal arcus using the merged datasets from the discovery and validation cohorts. The results revealed that 15 metabolites (mainly including FA species, short-chain CAR species, LPA species, and arachidonic acid-derived oxylipins) were associated with corneal arcus and xanthomas (Additional file 2: Table S4). After adjustments for age, sex, and clinical lipids, ten of them remained correlated with corneal arcus and xanthomas (as summarized in Fig. 3d). Notably, a panel of pro-inflammatory metabolites (including LPC 20:4, LPA 18:1, LPA 20:4, 11b-PGE2, and 15-oxoETE) were positively associated (β-coefficient values ranging from 0.328 to 0.653, all *P* values < 0.05; Additional file 2: Table S4).

### Metabolic alterations in arachidonic acid and lysophospholipid metabolism show significant associations with aortic stenosis

Aortic stenosis, including supravalvular aortic stenosis (SVAS) or calcific valvular aortic stenosis (VAS), is a major long-term complication of HoFH [18]. In regression analysis, our results demonstrated that LDL-C, TC, APOB, non-HDL, and Lp(a) showed positive correlations with SVAS (all *P* values < 0.05, Fig. 3c). Regarding to calcific VAS, we found that ages (β-coefficient = 0.161), male sex (β-coefficient = 0.528), and Lp(a) (β-coefficient = 0.469) was positively associated (all* P* values < 0.05), whereas LDL-C, TC, non-HDL, and APOB only showed a non-statistically positive correlation with VAS. Additionally, we found that HDL-C and APOA1 were negatively associated with SVAS and calcific VAS (Fig. 3c**)**.

Then, we performed univariate and multivariate regression analyses to explore the associations of HoFH-altered metabolites with SVAS and calcific VAS. A panel of inflammation-related metabolites was found to be positively associated with SVAS and calcific VAS (Additional file 2: Tables S5, S6). Our results demonstrated that six lysophospholipids (including LPC 20:4, LPC 18:0, LPE O-16:1, LPE O-18:0, LPA 20:4, and LPI 18:1) were significantly associated with SVAS (β-coefficient values ranging from 0.378 to 0.984, all *P* values < 0.05; Additional file 2: Table S5; Fig. 3e), even after adjustments for age, sex, and clinical lipids. In addition, our results indicated that arachidonic acid (FA 20:4), three LPA species (16:0, 18:0, and 18:2), and two arachidonic acid-derived oxylipins (11b-PGE2 and 15-HETE) were independently associated with calcific VAS (Additional file 2: Table S6; Fig. 3f). These findings indicated that the inflammation-related metabolites in the pathways of lysophospholipid and arachidonic acid metabolism might play a potential role in the progression of aortic stenosis.

### HoFH patients with and without ASCVD events show less heterogeneity in routine clinical risk factors

The most important characteristic in HoFH is premature ASCVD [10, 11]. Among the 232 HoFH individuals in discovery and validation cohorts, 37 patients had a history of ASCVD events. In the regression analysis of the associations between clinical lipids and ASCVD, the results demonstrated that Lp(a) was positively associated with the presence of ASCVD, whereas APOA1 and HDL-C were negatively associated (Additional file 2: Fig. S5a). To investigate the roles of clinical factors in predicting the first ASCVD event, a total of 84 HoFH patients (mean age: 25.6 ± 11.5 years; males: 53.1%) with available follow-up were enrolled from the discovery cohort (Fig. 1b). After a median follow-up of 4.9 years (interquartile range: 4.0–5.3 years), ASCVD events occurred in 33 patients, including 26 myocardial infarction and 7 cardiac death cases. The baseline clinical and genetic characteristics of the study subjects are summarized in Table 2. Patients who developed ASCVD were more likely to have at least one LDLR null mutation and exhibited higher levels of Lp(a) and lower baseline levels of HDL-C and APOA1. Of note, there were no significant differences in baseline LDL-C levels, mean LDL-C levels at follow-up, LDL-C year scores, and LLT measures between patients with ASCVD events and event-survived individuals (Table 2). The subsequent Cox regression proportional-hazards analyses found that Lp(a), HDL-C, APOA1, and LDLR null mutations were significantly related to incident ASCVD events (Additional file 2: Fig. S5b).

**Table 2:** Baseline clinical characteristics of 84 HoFH patients enrolled from the discovery cohort

	All subjects (*n* = 84)	No event subjects (*n* = 51)	Event subjects (*n* = 33)	*P* value
Ages	25.6 ± 11.5	24.1 ± 12.3	27.9 ± 9.8	0.13
Male sex, *n* (%)	43 (53.1%)	27 (52.9%)	16 (48.5%)	0.16
BMI, kg/m^2^	20.6 ± 1.8	20.3 ± 1.9	21.1 ± 1.4	0.08
Hypertension, *n* (%)	4 (4.8%)	3 (5.9%)	1 (3%)	0.55
Diabetes mellitus, *n* (%)	0 (0%)	0 (0%)	0 (0%)	
Active smoking, *n* (%)	0 (0%)	0 (0%)	0 (0%)	
At least one null LDLR, *n* (%)	52 (61.9%)	27 (52.9%)	25 (75.8%)	0.04
True HoFH, *n* (%)	26 (31%)	17 (33.3%)	9 (27.3%)	0.56
Xanthomas, *n* (%)	45 (53.6%)	25 (49%)	20 (60.6%)	0.3
Corneal arcus, *n* (%)	39 (46.4%)	20 (39.2%)	19 (57.6%)	0.1
Clinical lipid profiles				
Baseline LDL-C, mmol/L	13.03 ± 4.13	12.94 ± 4.45	13.17 ± 3.63	0.82
Baseline TC, mmol/L	15.53 ± 4.50	15.61 ± 5.01	15.41 ± 3.62	0.84
Baseline TG, mmol/L	0.92 [0.70, 1.49]	0.86 [0.67, 1.42]	1.1 [0.75, 1.55]	0.46
Baseline HDL, mmol/L	0.82 [0.61, 1.12]	0.99 [0.72, 1.31]	0.7 [0.47, 0.89]	0.001
Baseline Lp(a), mg/dL	47.3 [28.5, 81]	40.9 [25.8, 56]	72.1 [46.3, 95]	0.001
Baseline APOA1, g/L	0.79 ± 0.26	0.85 ± 0.23	0.72 ± 0.26	0.01
Baseline APOB, g/L	2.5 ± 0.73	2.45 ± 0.73	2.57 ± 0.75	0.73
LDL-C at last visit, mmol/L	10.47 ± 2.97	10.21 ± 3.27	10.89 ± 2.41	0. 31
Mean LDL-C at follow-up, mmol/L	10.78 ± 2.79	10.41 ± 3.02	11.37 ± 2.33	0.12
LDL-C year score, mmol/L-year	359.2 [235.2, 511.7]	296.4 [212.9, 462.7]	448 [302.8, 556.1]	0.07
LLT status				
Ages at initiation of LLT	12.3 ± 7.9	11.9 ± 8.6	12.7 ± 6.7	0.66
Statin + Probucol, *n* (%)	6 (7.1%)	4 (7.8%)	2 (6.1%)	0.76
Stain + Ezetimibe, *n* (%)	75 (89.3%)	43 (84.3%)	32 (97.0%)	0.067
Stain + Ezetimibe + PCSK9 inhibitor, *n* (%)	3 (3.6%)	2 (3.9%)	1 (3.0%)	0.83

Continuous data are presented as mean ± standard deviation or median [interquartile range], and categorical variables are presented as %. Two-tailed Student’s *t* test or Mann Whitney *U* test were used for continuous data. The chi-square test was used for categorical data

*BMI* body mass index, *CVD* cardiovascular disease, *LLT* lipid-lowering therapy, *LDL-C* low-density lipoprotein cholesterol, *TC* total cholesterol, *TG* triglycerides, *HDL-C* high-density lipoprotein cholesterol, *APOB* apolipoprotein B, *APOA1* apolipoprotein A1, *Lp(a)* lipoprotein (a)

### Targeted metabolomics identifies significant metabolic alterations in HoFH patients with ASCVD events

Next, univariate and multivariate regression analyses were performed to investigate the associations of circulating metabolites with the presence of ASCVD in all studied HoFH patients. The results demonstrated that a panel of metabolites in the pathways of arachidonic acid metabolism, fatty acid metabolism, phospholipid metabolism, and carnitine metabolism were closely related to ASCVD (Additional file 2: Table S7). Furthermore, most of these metabolites were implicated in the processes of inflammation, oxidative stress, and atherosclerosis (Fig. 3b). We hypothesized that the serum metabolites might be used as cardiovascular risk factors for predicting ASCVD events in HoFH patients. In the untargeted metabolomic dataset of 84 HoFH patients in the follow-up study, a small panel of 22 chemical standard-annotated metabolites were identified to be differentially expressed at baseline (FDR-adjusted *P* value < 0.05) between subjects who developed ASCVD events and patients without events at follow-up (Additional file 2: Fig. S6). These altered metabolites are mainly dominated by lipid alterations in the pathways of fatty acid and carnitine biosynthesis, arachidonic acid metabolism, and phospholipid metabolism (Additional file 2: Table S8).

**Fig. 4:**
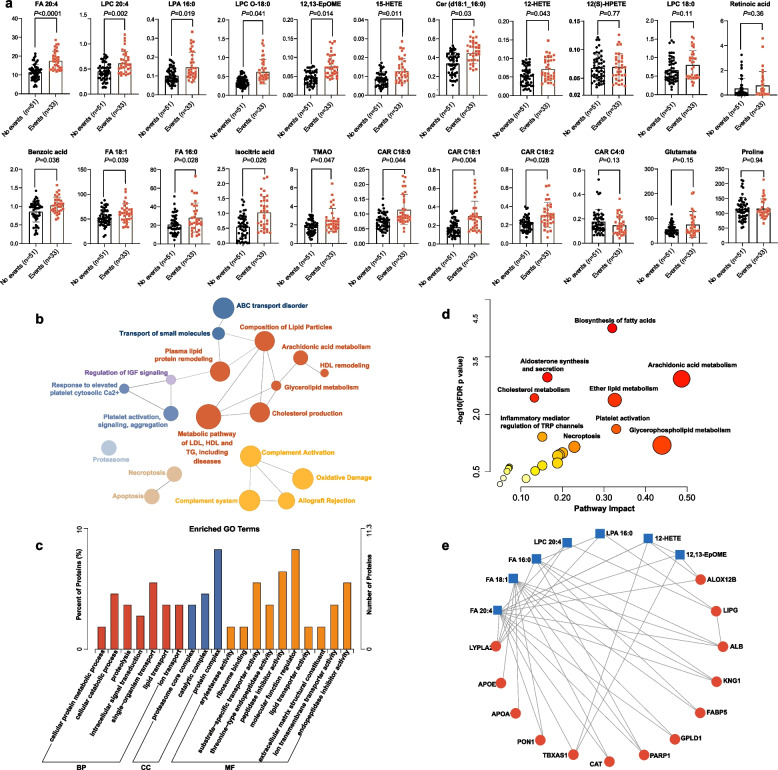
ASCVD-associated metabolite and protein alterations in the sera of HoFH patients. **a** Dot histogram of metabolite levels in HoFH patients with and without ASCVD events (concentration, nmol/mL). Mann–Whitney *U* test were used for each comparison. **b** Functionally grouped network of enriched pathways of differentiated proteins in patients with ASCVD events. **c** Enriched GO categories of differentiated proteins. BP biological process, CC cellular component, MP molecular function. **d** Topology analysis of pathway enrichment and impact from the joint pathway of differentially expressed proteins and metabolites. **e** Metabolite-protein interaction network. **f** Topology analysis of pathway enrichment and impact from the joint-pathway of differentially expressed proteins and metabolites. LPA 16:0 lysophosphatidic acid 16:0, LPC-O 18:0 lysophosphocholine alkyl − 18:0, LPC 20:4 lysophosphocholine 20:4, 12,13-EpOME 12,13-epoxyoctadecenoic acid, Cer (d18:1_16:0) Ceramide (d18:1_16:0). Other abbreviations are seen in Figs. 1 and 2

To verify the qualitative and quantitative accuracy for these altered metabolites, MRM-based targeted quantification and Pearson correlation analysis were performed. The quantitative levels of 22 metabolites were summarized in Fig. 4a. In the correlation analysis, the quantitative values of 20 metabolites showed a high correlation with their semi-quantitative levels obtained from untargeted metabolic profiling (correlation *r* coefficients ranging from 0.43 to 0.85, all *P* values < 0.001; Additional file 2: Fig. S7a). We also performed Spearman’s rank correlation and debiased sparse partial correlation network to explore the associations between the 22 differentiated metabolites. From the resultant correlograms (Additional file 2: Fig. S7 b, c), it was evident that most of these differentiated metabolites correlated with each other and acted in a coordinated manner. Furthermore, Cox regression analysis revealed that LDLR null mutations were positively associated with ASCVD events (Additional file 2: Fig. S5b). Interestingly, among the 52 patients with at least LDLR null mutation, we found that patients who developed ASCVD (*n* = 25) were more likely to have two LDLR null mutations (Additional file 2: Table S9) and presented higher sera concentrations of FA species, LPC species, CAR species, and arachidonic acid-derived oxylipins than those without ASCVD events (*n* = 27).

### Integration of deep proteomics and metabolomics reveals a complex ASCVD-associated metabolic network

Next, we used a deep proteomic strategy with multi-nanoparticle protein corona to investigate the ASCVD-associated proteins and constructed the ASCVD-associated metabolic association network at multi-omic levels. Altogether, a total of 2304 proteins were identified and quantified. We found that 142 proteins were differentially expressed in the sera of patients with and without ASCVD events (Additional file 2: Fig. S8 a, b). According to the distribution of subcellular localization (Additional file 2: Fig. S8c), these differentiated proteins mainly included extracellular proteins (43.36%), nucleus proteins (16.78%), and cytoplasm proteins (15.38%). Pathway network and Gene Ontology (GO) enrichment analyses demonstrated that these differentiated proteins were mainly involved in the biological processes of small-molecular lipid transport and metabolism (e.g., arachidonic acid, glycerolipid, and cholesterol metabolism), lipid protein remodeling and related metabolism, platelet activation, and complement activation (Fig. 4 b, c).

To investigate the common metabolic pathways of differentially expressed proteins and metabolites, the joint-pathway enrichment and integration network analyses was performed based on MetaboAnalyst. The resultant plot revealed that arachidonic acid metabolism, glycerophospholipid metabolism, fatty acid synthesis, platelet activation, aldosterone synthesis, and ether lipid metabolism were significantly enriched (Fig. 4d). The integration network of key pathway-based metabolites and proteins is shown in Fig. 4e, including 13 proteins and seven metabolites.

### Random Forest algorithm identifies a panel of metabolic/clinical features that shows significant performances in predicting ASCVD events

**Fig. 5:**
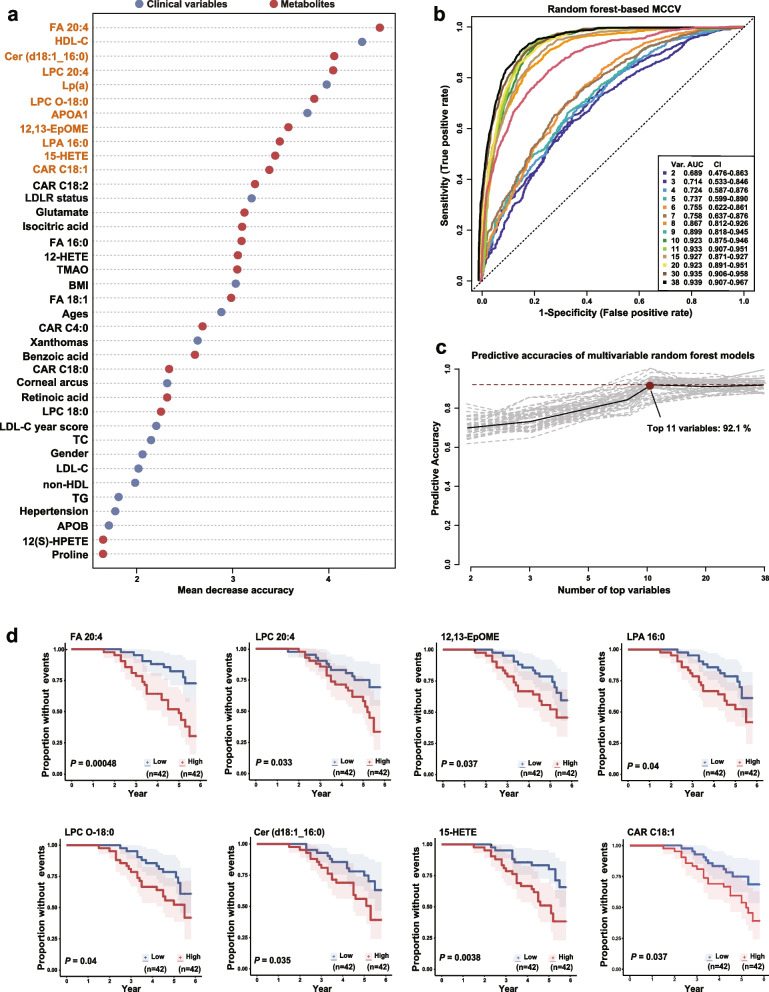
Selection of the optimal ASCVD-related metabolic/clinical variable panel by using Random Forest algorithm. **a** Top important ASCVD-associated variable selection by using the values of mean decrease accuracy in Random Forest analysis. **b** ROC curves generated from MCCV-based multivariate Random Forest models. The AUC-ROC value and its 95% CI are shown. **c** Predictive accuracies of multivariable Random Forest models with different numbers of top important variables. **d** Kaplan–Meier survival analysis of eight top important metabolites in HoFH patients with and without ASCVD events during the follow-up. Log-rank test *P* values are shown. LDLR status: at least one null mutation. Other abbreviations are shown in Table 2 and Figs. 2 and 3

To select a small panel of important metabolic/clinical markers that can maintain a maximized performance in predicting ASCVD events, Random Forest (RF) algorithm was performed based on the integrated datasets of 22 differentiated metabolites and 16 clinical variables from the 84 HoFH patients. The variable importance of each metabolite and clinical measure was ranked by using the values of mean decrease accuracy and summarized in Fig. 5a. Then, the Monte Carlo cross validation (MCCV) analysis based on multivariable RF models was performed to select the optimal number of important variables. As shown in Fig. 5b and c, the combination of the top eleven features, including three clinical lipids (HDL-C, Lp(a), and APOA1) and eight metabolites (FA 20:4, LPC O-18:0, LPA 16:0, LPC 20:4, Cer (d18:1_16:0), 12,13-EpOME, 15-HETE, and CAR C18:1) and showed the powerful performance in predicting ASCVD events (AUC-ROC value = 0.933; predictive accuracy = 92.1%), while the additional features had little effect on the values of AUC-ROC and predictive accuracy (Fig. 5b, c). Notably, most of these eight metabolites were closely associated with inflammatory processes and atherosclerosis procession (as shown in Fig. 3b). Furthermore, Kaplan–Meier curve analysis (Fig. 5d) indicated that HoFH individuals with baseline concentrations of the top eight metabolites in the upper half had lower probability of ASCVD event-free survival than patients in the lower half (log-rank test *P* values ranging from 0.04 to 0.00048).

## Discussion

To our knowledge, this is the first study to describe the comprehensive metabolome and lipidome maps in HoFH patients compared to HeFH and non-FH individuals. The main findings of the present study are as follows: First, HoFH patients were characterized by significant metabolite perturbations that mapped to various metabolic metabolism, inflammation, and oxidative stress. Second, Lp(a) and a panel of inflammatory metabolites were closely related to corneal arcus, xanthomas, and aortic stenosis. Third, we identified a small panel of metabolomic alterations and clinical lipids that exhibited significant predictive performance on ASCVD events in patients with HoFH.

Compare with patients with HoFH, patients with HoFH can develop premature ASCVD events and have higher prevalences of extensive xanthomas, corneal arcus, and aortic stenosis. Unlike the HeFH population for which multiple biochemical variables and robust cardiovascular risk prediction models are available [37–39], molecular information and risk stratification in patients with HoFH had been mostly limited to information on the LDL-C levels, LLT measures, and genotypes [13, 14, 40]. In this study, we collected the datasets of comprehensive metabolomic and lipidomic profiles from the largest multi-center panel of HoFH patients across mainland China since 2003. To be able to extract valuable information from complex metabolomic datasets, supervised/unsupervised MVA and multivariable regression analyses were performed to explore the metabolomic landscape of HoFH and identify the clinical phenotype-associated metabolite markers. Compared with the commonly used classification/discrimination methods such as univariate statistical analysis, these statistical approaches could capture the key metabolic pattern-associated features in the complex high dimensional datasets.

Our metabolic findings indicated a significant enrichment in the biosynthesis pathways of steroids, fatty acids, and carnitines (Fig. 3a). Free cholesterol and cholesterol ester (CE) are the major basic elements of LDL-C and TC [41]. Our metabolomic data indicated a generalized exaltation of free cholesterol and four CE species containing FA (16:0, 16:1, 18:0, 18:1) from de novo fatty acid biosynthesis pathway in the sera of patients with HoFH. In addition, our results demonstrated that these free saturated/monounsaturated FA species and their acyl carnitine (CAR) forms in the sera of HoFH patients were significantly higher than those in HeFH patients and non-FH individuals. Saturated/monounsaturated long-chain FA and acyl-CAR species are toxic. Previous evidence has revealed that their accumulation might lead to mitochondrial dysfunction and promote atherosclerosis progression [42, 43]. Our results also demonstrated that FA and acyl-CAR species were up-regulated in HoFH patients who developed ASCVD events (Fig. 3a). These findings suggested that an upregulation of de novo FA biosynthesis in HoFH might contribute to toxic CE and CAR overload, additionally leading to the development of ASCVD.

This study also demonstrated that HoFH patients were characterized by significant perturbations in the pathways of phospholipid and bile acid biosynthesis (Fig. 3 and Additional file 2: Table S3). We found that HoFH patients showed higher serum levels of LPC, LPC-O, and primary bile acids (cholic acid and glycocholic acid) than HeFH and non-FH individuals. Previous animal studies indicated that LPC and primary bile acids could induce a significant elevation of cholesterol absorption and increase blood cholesterol [44, 45]. These evidences suggested the HoFH-perturbed LPC and bile acids expressions might play important roles in cholesterol homeostasis, providing potential LLT-targets for HoFH. LPC and LPC-O are important basic elements of LDL-C and oxidized LDL-C. Previous studies also indicated that these two lysophospholipids could induce inflammatory responses and oxidative stress [46]. Interestingly, several LPC and LPC-O species were negatively associated with endogenous anti-inflammatory and anti-oxidative lipoproteins, including HDL-C and APOA1 (Additional file 2: Fig. S4). Deep proteomics indicated that the upstream regulator of LPC, namely LYPLA2, was increased in the serum of patients with ASCVD events (Fig. 4; Additional file 2: Fig. S8). Furthermore, RF analyses indicated that the increased baseline levels of LPC and LPC-O species were significantly associated with ASCVD events in HoFH patients. These results suggested that perturbed phospholipid metabolism might also play important roles in the progression of ASCVD.

Another important finding from this study was that HoFH patients showed profound perturbations in the pathways of arachidonic acid metabolism and sphingolipid metabolism compared to HeFH and non-FH subjects (Fig. 3a; Additional file 2: Table S3). Ceramide (Cer), the bioactive metabolite in the pathway of sphingolipid metabolism, could accumulate in atherosclerotic lesions [47], and the higher circulating levels of Cer displayed strong predictive value of plaque instability and future adverse cardiovascular events that went far beyond LDL-C [47, 48]. Numerous studies have demonstrated the negative functions of arachidonic acid (FA 20:4) and its oxidative metabolism derivatives (oxylipins) in regulating atherogenesis, inflammation, and thrombosis, thus holding a critical role in the emergence and progression of cardiovascular diseases [49, 50]. In this study, we found that the increased levels of oxylipins were significantly correlated with the decreased levels of anti-inflammatory HDL-C and APOA1. The Kaplan–Meier curve analysis (Fig. 5d) demonstrated that HoFH patients with baseline concentrations of FA 20:4 and two oxylipins (15-HETE and 12,13-EpOME) in the upper half had lower probability of ASCVD event-free survival than individuals in the lower half. Furthermore, the proteomic analysis revealed that LYPLA2 and ALOX12B were elevated in patients with ASCVD events (Additional file 2: Fig. S8). These two proteins play crucial roles in the release of FA 20:4 from phospholipids and the synthesis of oxylipins from FA 20:4 [51].

Excess cholesterol undoubtedly plays a causing role in promoting the formation of xanthomas and corneal arcus. Interestingly, we found that LDL-C and TC only showed a non-statistically positive correlation trend with the presences of xanthomas and corneal arcus in patients with HoFH (Fig. 3). Alternatively, our data demonstrated that Lp(a), three lysophosphatidic acid (LPA) species (LPA 18:1, LPA 18:2, LPA 20:4), and four oxidative metabolites of arachidonic acid (including 12(S)-HPETE, 12-HETE, 15-oxoETE, and 11b-PGE2) showed positive associations with corneal arcus and xanthomas. Previous studies revealed that inflammation was a pathogenetic factor of xanthomas [52]. LPA is the end-product of Lp(a) metabolism [53]. Like arachidonic acid-derived oxylipins, the pro-inflammatory properties of Lp(a) and LPA have also been well documented [54, 55].

The high prevalence of aortic stenosis is another major cause of mortality in HoFH patients [56]. Although the risk of aortic stenosis and specifically SVAS in the long-term statin era was decreased, the risk of calcific VAS remained high. Moreover, VAS progression cannot be affected by LLT, even with normalization of cholesterol levels [9, 18]. Interestingly, our results indicated that LDL-C and TC only showed a significant correlation with SVAS, but not calcific VAS. Sex, ages, and Lp(a) were found to be significantly associated with calcific VAS (Fig. 3). At metabolomic level, our findings demonstrated that three LPA species (16:0, 18:0, 18:2), arachidonic acid, and its oxidative metabolites (15-HETE and 11b-PGE2) were independently correlated with calcific VAS. These findings suggested that elevated metabolic productions from the Lp(a) and arachidonic acid metabolism pathways together with extremely elevated cholesterol might synergistically contribute to the formation of corneal arcus, xanthomas, and calcific VAS in HoFH patients.

Although this study highlighted new molecular evidences of unknown origin in human HoFH. Several potential limitations deserve closer attention. First, the ethnic homogeneity of our study population might limit the generalizability of our findings to other populations. Second, future validation of the ASCVD prediction accuracy of the identified metabolomic/clinical marker panel in external and larger HoFH cohorts is warranted. Third, our proteomics study had a limited number of patients, and future studies on investigating the altered proteins in a large panel of HoFH patients will offer great promise for the comprehensive understanding of molecular perturbations in human HoFH. Fourth, additional data will be needed to explore the source of HoFH-associated circulating metabolites and identify the pathophysiological roles of key metabolites in the development of cardiovascular complications.

## Conclusions

Collectively, this study demonstrated that human HoFH was associated with diverse metabolic repercussions and was more complex than previously known. Importantly, we identified a variety of bioactive and functional metabolites that were closely associated with the aggressive clinical phenotypes and cardiovascular complications in patients with HoFH. Our findings offer new mechanistic avenues to better understand the cholesterol dysregulation, aortic stenosis, and atherosclerosis progression of HoFH and may help in providing future directions for developing novel therapeutic strategies and accurate ASCVD risk prediction models that might benefit HoFH patients.

## Supplementary Information

**Additional file 1. **Supplementary methods.

**Additional file 2: Fig. S1. **Stability assessment of untargeted metabolomic data from the discovery and validation cohorts. **Fig. S2.** Assessment and establishment of PLS-DA models for discriminating groups and identifying differentiated metabolites in discovery cohort. **Fig. S3.** PLS-DA model validation of the differentially expressed metabolites for discriminating HoFH from non-FH and HeFH in validation cohort. **Fig. S4.** Correlation pattern analyses between discriminatory metabolites and clinical features. **Fig. S5.** The association of clinical/genetic risk factors with ASCVD.** Fig. S6.** Volcano plot of metabolite variations in pairwise comparisons of events vs. non-events. **Fig. S7.** Correlations between ASCVD-associated metabolomic markers. **Fig. S8.** Differentiated proteins identified in the comparison of patients with and without ASCVD events. **Table S1.** Demographic and clinical characteristics of all subjects in the validation cohort. **Table S2.** Detailed information on lipid-lowering therapies in both cohorts. **Table S3.** Metabolites for differentiating HoFH from HeFH and non-FH in the discovery cohort. **Table S4.** Associations between sera metabolites and corneal arcus/xanthomas by using regression analyses. **Table S5.** Associations between serum metabolites and supravalvular aortic stenosis by using regression analyses. **Table S6.** Associations between serum metabolites and calcific valvular aortic stenosis by using regression analyses. **Table S7.** Associations between serum metabolites and the presence of ASCVD by using regression analyses. **Table S8.** Differentiated metabolites identified in the comparison of HoFH patients with and without ASCVD events. **Table S9. **Concentrations of ASCVD-associated metabolites in HoFH patients with at least one LDLR mutation.

**Additional file 3. **The annotated metabolites in the metabolomics and lipidomics analysis.

**Additional file 4. **List of detailed metabolites contributing to the enriched metabolic pathway.

## Data Availability

Raw metabolomic data have been deposited at https://service.most.gov.cn/ and are available from the corresponding authors (L.Y. Wang and Y.W. Qin) and the manager of National Key Research and Development Program of China upon request. Any additional information required to reanalyze the data reported in this paper is available from the corresponding authors on reasonable request.
