# Supplementary material for: Metabolic systems approaches update molecular insights of clinical phenotypes and cardiovascular risk in patients with homozygous familial hypercholesterolemia
Source: BMC Med. 2023 Jul 27;21:275. doi: 10.1186/s12916-023-02967-8 (PMC10375787; doi:10.1186/s12916-023-02967-8)
Supplement: Supplementary file 1 — Additional file 1. Supplementary methods. [file 12916_2023_2967_MOESM1_ESM.docx]

**Additional file 1.**

**Supplementary Methods**

**High‑performance liquid chromatography condition**

For the aqueous phase (untargeted metabolomics), the hydrophilic residues were suspended with 100 μL of acetonitrile: water (1:1, v/v) solution. The separation of metabolites was performed on a Xbridge amide column (100 × 2.1 mm, 3.5 μm; Waters Corp., Milford, MA, USA). The optimal mobile phase consisted of a linear gradient system of phase A (5 mM ammonium acetate in water with 5% acetonitrile), and mobile phase B (acetonitrile): 0 min, 95% B; 3 min, 90% B; 13 min, 50% B, 14 min, 50% B; 15 min, 95% B; 17 min, 95% B. For the hydrophobic phase (untargeted lipidomics), the lipid residues were reconstituted in 100 μL of chloroform: methanol (1:1, v/v) solution and then diluted threefold with isopropanol: acetonitrile: water (2:1:1, v/v/v) solution. The separation of hydrophobic metabolites was performed on a reversed phase X-select CSH C18 column (2.1 mm × 100 mm, 2.5 μm; Waters Corp., Milford, MA, USA). The optimal mobile phase consisted of a linear gradient system of phase A (10 mM ammonium acetate and 0.1% formic acid in acetonitrile with 40% water), and mobile phase B (isopropanol: acetonitrile, 9:1, v/v): 0 min 40% B; 2 min 43% B; 12 min 60% B; 12.1 75% B; 18 min 99% B; 19 min 99% B; 20 min 40% B. The column temperature was maintained at 40 °C and the flow rate remained constant at 0.4 mL/min. The sample injection volume of each sample was 10 μL.

**Q-Exactive HF Mass spectrometry condition**

The parameters of the mass spectrometry detection were followed as previously described [30,31]. All analyses were based on data-dependent acquisition (DDA) model using the Q-Exactive HF MS (Thermo Fisher Scientific, Waltham, MA, USA), Acquisition was performed on positive and negative ion modes. The spray voltage was 3.5 kV for positive ion mode (ESI+) and 3.0 kV for negative ion model (ESI−); Each acquisition cycle consists of one survey scan (MS1) at 60,000 resolutions from 50 to 900 *m/z* for the hydrophilic metabolites and 200 to 1200 *m/z* for the hydrophobic metabolites, followed by 10 MS/MS scans in HCD mode at 15,000 resolutions using step-NCE of 15, 30 and 45. The dynamic exclusion was set to 10 s, sheath gas was 40 L/h; aux gas was 10 L/h. The probe heater temperature and capillary temperature were set as 300 °C and 320 °C, respectively. To monitor the robustness of sample pretreatment and stability of LC-MS analysis, the QC samples were analyzed between every 20 samples during the metabolomic and lipidomic analyses.

**Data** **processing and metabolite identification**

All the LC-MS acquired metabolomic and lipidomic data was transformed to MS-DIAL software v3.6 for deconvolution, alignment, and data reduction by using the QC samples. Then, a list of raw data matrix including information of precursor ions, fragment ions, neutral molecules, and intensity was generated for further analysis. The raw data matrix was normalized by the QC samples and the internal standards using MetaboDrift software as previously described. The normalized metabolite features that were absent in more than 10% of pooled QC injections throughout analysis were removed. From the remaining features those with more than 20% relative standard deviation (RSD) in peak intensity across pooled QC injections were also removed. Positive and negative data sets were merged. The MS1 and MS2 spectra-based metabolite identification was performed in MS-DIAL software, Progenesis QI (Waters, Manchester, U.K.), and by searching the acquired spectra against the MassBank database, LipidBlast-based in silico spectra database, QI MetaScope database, METLIN database, HMDB database, and LIPIDMAPS database, using a retention time shift ± 0.3min and accurate mass ± 5 ppm filters. Furthermore, the identified metabolites were also confirmed by using our in-house metabolite library, which contains of the retention time, accurate mass, and MS/MS spectra of commercially available metabolite standards. Metabolites identified as xenobiotics were excluded from the analysis.

**MRM-based targeted metabolites analysis**

To obtain a more accurately qualitative and quantitative analysis of the identified metabolic signatures from untargeted metabolomic data for cardiovascular risk prediction studies, MRM-based metabolite analysis was detected by Metware Ltd. (http://www.metware.cn/) based on the AB Sciex QTRAP 6500 LC-MS/MS platform. The sample preparation, chromatographic column, mobile phase, and column temperature conditions were referred to those in the untargeted metabolomic profiles. The flow rate of the mobile phase remained constant at 0.4 mL/min. Both positive and negative polarities were adopted. Ion source parameters were optimized as follows: ion spray needle voltage, 5500 V/− 4500 V; GS1, GS2, and CUR were set as 45 psi, 45 psi, and 35 psi, respectively; turbo gas temperature, 550 °C; collisional activated dissociation (CAD) gas, medium level.

Delustering potential (DP) and collision energy (CE) for individual MRM transitions was done with further DP and CE optimization. A specific set of MRM transitions were monitored for each metabolite as followed: 12(S)-HPETE (317.1→153.0; DP: -90; CE: -20), 12,13-EpOME (295.0→195.0; DP: -90; CE: -30), 12-HETE (319.0→135.0; DP: -90; CE: -16), 15-HETE (319.0→175.0; DP: -85; CE: -19), benzoic acid (121.1→77.1; DP: -65; CE: -30), CAR C18:0 (428.3→85; DP: 50; CE: 24), CAR C18:1 (426.3→85; DP: 50; CE: 26), CAR C18:2 (424.3→85; DP: 50; CE: 26), CAR C4:0 (232.1→85; DP: 25; CE: 10), Cer (d18:1_16:0) (538.4→520; DP: 90; CE: 10), FA 16:0 (255.1→96.8; DP: -110; CE: -30), FA 18:1 (281.1→97; DP: -67; CE: -33), FA 20:4 (303.2→259; DP: -110; CE: -19), glutamic acid (146.0→128; DP: -50; CE: -15), isocitric acid (191→73; DP: -30; CE: -20), LPA 16:0 (409→255; DP: -170; CE: -30), LPC 18:0 (524.4→184.1; DP: 100; CE: 15), LPC 20:4 (544.4→184.1; DP: 100; CE: 20), LPC-O 18:0 (510.4→184.1; DP: 70; CE: 30), proline (115.8→69.8; DP: 30; CE: 12), retinoic acid (299→135; DP: -80; CE: -36), trimethylamine N-oxide (75.8→58.7; DP: 20; CE: 14). The quantitative values of each metabolite were calculated by the isotope labeled internal standard (arachidonic acid-*d*_5_ LPC (19:0)-*d*_5_, (±)15-HETE-*d*_8_, stearoyl-L-carnitine-*d*_3_, Cer-d7 (d18:1 /16:0)-*d*_7_, palmitoyl-*d*_9_ LPA, and L-phenyl-*d*_5_-alanine). QC samples were measured every six samples. The coefficients of variation (CV) of QC samples were calculated by the ratios of standard deviations / means and the CV values were listed in the Table S9. The CV values of all determined metabolites in QC samples were less than 0.2, indicating a good stability during collection process.

**Protein corona preparation and data-independent acquisition-based deep proteomics analysis**

Deep proteomics analysis was detected by Novogene Co., Ltd. (https://www.novogene.com/) based on the Thermo Fisher Q Exactive TM HF-X LC-MS/MS platform. To form the protein corona, a total of 40 μL sera was added with equal volumes of wash buffer and DI water containing of nano magnetic beads. The mixture was sealed and incubated at 37 °C for 1 h with shaking at 300 rpm for 1 hour. The supernatant was removed by magnetic separation, the protein corona was further washed with 3 times volume of wash buffer for 5 min. To digest the proteins bound onto nano magnetic beads, a trypsin digestion kit was used according to previously reported protocols [32].

For data-independent acquisition (DIA) analyses using EASY-nLC^TM^-HFX, mobile phases A (0.1% FA in H2O) and B (0.1% FA in 80% ACN) were used to develop a gradient elution. A half sample containing 4 μg fraction supernatant and 0.8 μL iRT reagent was injected into the EASY-nLCTM 1200 UHPLC system coupled with an Orbitrap Q Exactive^TM^ HF-X mass spectrometer operating in the data-independent acquisition (DIA) mode with spray voltage of 2.1 kV, Nanospray Flex™ (ESI), and capillary temperature of 320°C. For DIA acquisition, the *m/z* range covered from 350 to 1500. MS1 resolution was set to 60000 (at m/z 200), full scan AGC target value was 5×105, the maximum ion injection time was 20 ms. Peptides were fragmented by HCD in MS2, in which resolution was set to 30000 (at 200 m/z), AGC target value was 1×106, a normalized collision energy of 27%. The DIA data was imported into Spectronaut software to generate a DDA library and ion-pair chromatographic peaks were extracted. Matching the ion and calculating peak area to achieve the qualitative and quantitative of peptides. The iRT was added to the sample for correcting retention time, and the precursor ion Q value cutoff was set to 0.01. Proteins with fold-change beyond 1.5 or below 0.67 with adjusted p value lower than 0.05 were considered as significantly differential expression.
