# Supplementary material for: Metabolic systems approaches update molecular insights of clinical phenotypes and cardiovascular risk in patients with homozygous familial hypercholesterolemia
Source: BMC Med. 2023 Jul 27;21:275. doi: 10.1186/s12916-023-02967-8 (PMC10375787; doi:10.1186/s12916-023-02967-8)
Supplement: Supplementary file 2 — Additional file 2: Fig. S1. Stability assessment of untargeted metabolomic data from the discovery and validation cohorts. Fig. S2. Assessment and establishment of PLS-DA models for discriminating groups and identifying differentiated metabolites in discovery cohort. Fig. S3. PLS-DA model validation of the differentially expressed metabolites for discriminating HoFH from non-FH and HeFH in validation cohort. Fig. S4. Correlation pattern analyses between discriminatory metabolites and clinical features. Fig. S5. The association of clinical/genetic risk factors with ASCVD. Fig. S6. Volcano plot of metabolite variations in pairwise comparisons of events vs. non-events. Fig. S7. Correlations between ASCVD-associated metabolomic markers. Fig. S8. Differentiated proteins identified in the comparison of patients with and without ASCVD events. Table S1. Demographic and clinical characteristics of all subjects in the validation cohort. Table S2. Detailed information on lipid-lowering therapies in both cohorts. Table S3. Metabolites for differentiating HoFH from HeFH and non-FH in the discovery cohort. Table S4. Associations between sera metabolites and corneal arcus/xanthomas by using regression analyses. Table S5. Associations between serum metabolites and supravalvular aortic stenosis by using regression analyses. Table S6. Associations between serum metabolites and calcific valvular aortic stenosis by using regression analyses. Table S7. Associations between serum metabolites and the presence of ASCVD by using regression analyses. Table S8. Differentiated metabolites identified in the comparison of HoFH patients with and without ASCVD events. Table S9. Concentrations of ASCVD-associated metabolites in HoFH patients with at least one LDLR mutation. [file 12916_2023_2967_MOESM2_ESM.docx]

**Additional file 2.**


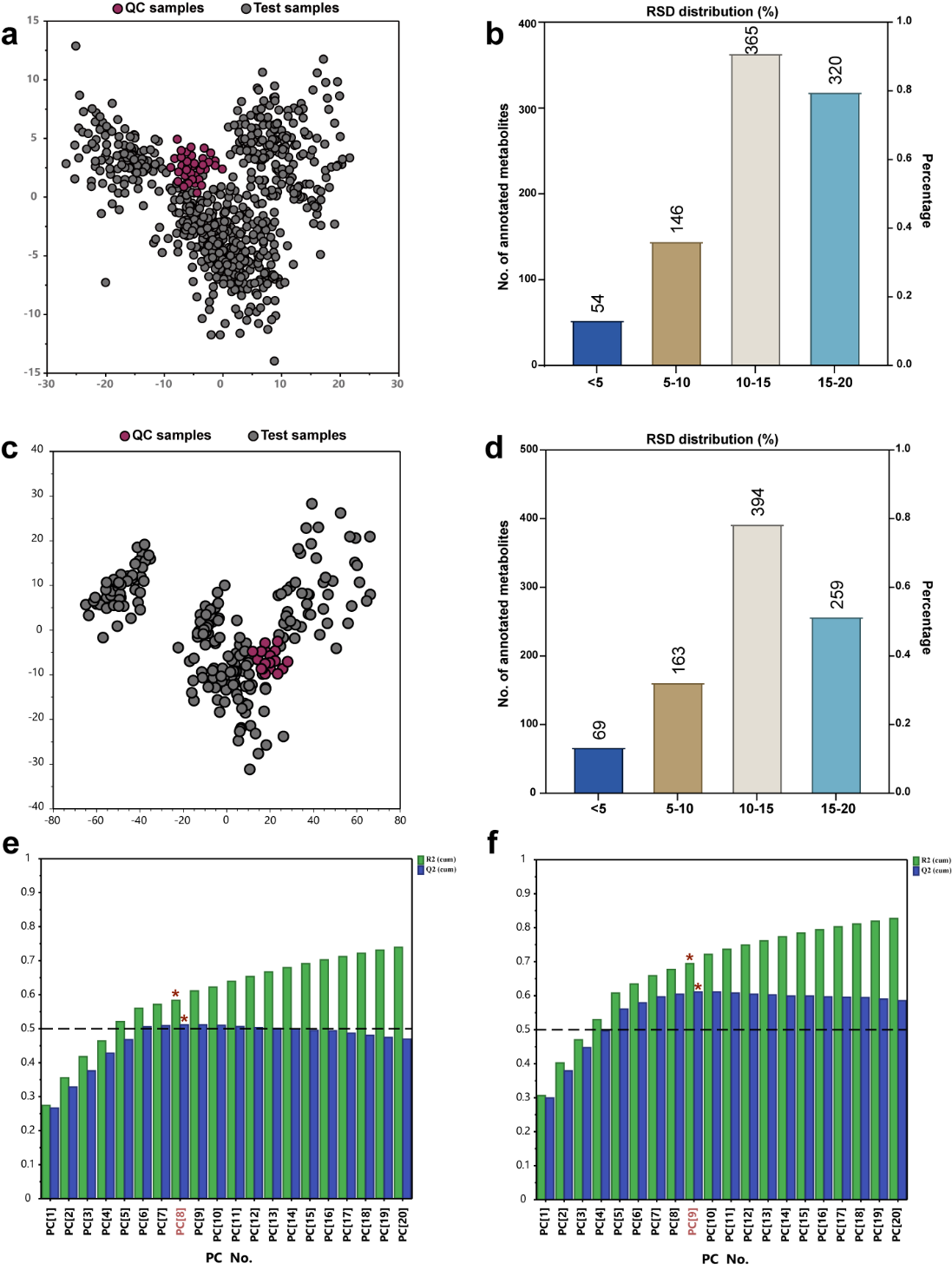


**Fig. S1 Stability assessment of untargeted metabolomic data from the discovery and validation cohorts.** **(a, b)** depicting PCA scores plot of all test samples and QC samples from the discovery cohort and the relative standard derivations (RSDs) of the distribution for the identified metabolites in the QC samples. **(c, d)** depicting PCA scores plot of all test samples and QC samples from the validation cohort and the RSDs of the distribution for the identified metabolites in the QC samples. **(e, f)** depicting the plot of cumulative Q2 and R2 values for selecting the optimized number of PCs in the establishment of PCA models of the discovery and validation cohorts, respectively. The cumulative R2 value >0.5 and Q2 value >0.5 indicated a good explanatory and predictive ability of the established PCA models.


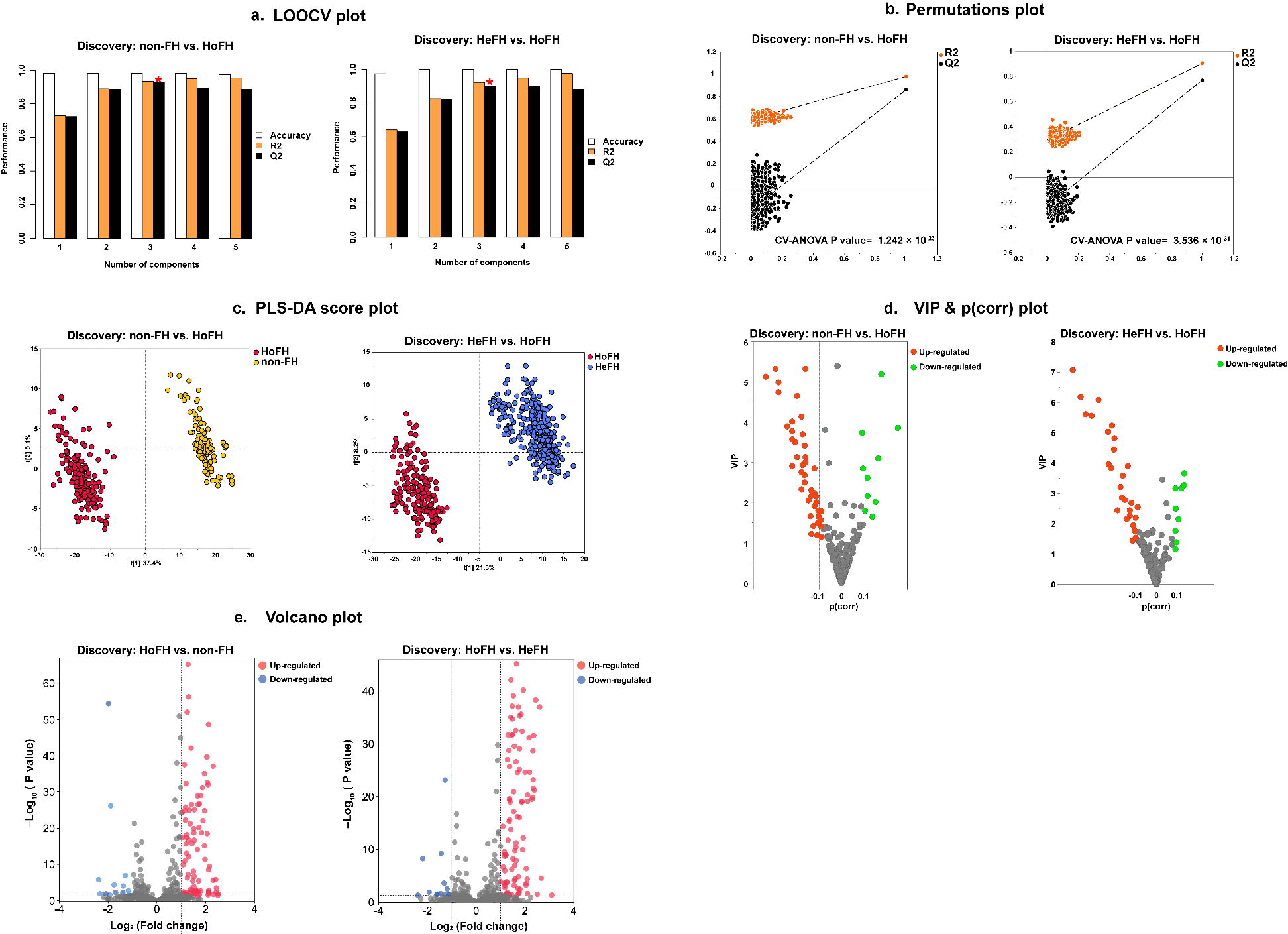


**Fig. S2** **Assessment and establishment of PLS-DA models** **for discriminating groups and identifying differentiated metabolites in discovery cohort.** **(a)** performance measure plots of leave-one-out cross validation (LOOCV). Q2 value > 0.4 in the first component was considered as a reliable model, all the LOOCV plots showed that the three-component model was best (indicated by a red star). **(b)** plots of permutation test. The criteria for PLS-DA model validity: All Q2-values to the left are lower than the original points to the right, the left regression line of the Q2-points intersects the vertical axis (on the left) at, or below zero; all R2-values to the left are lower than the original point to the right; CV-ANOVA *P* value <0.05. **(c)** two components-based PLS-DA scores plot of the metabolomic data; each point represents an individual serum sample. **(d)** VIP and p(corr) plots. The whole set of metabolites are ranked according to their VIP values, *p* (corr) values. VIP > 1 or absolute *p* (corr) values > 0.1 represents a significant importance of the metabolic variables in differentiating groups. **(e)** Volcano plots of each metabolite variation in pairwise comparisons of HoFH/non-FH and HoFH/HeFH.


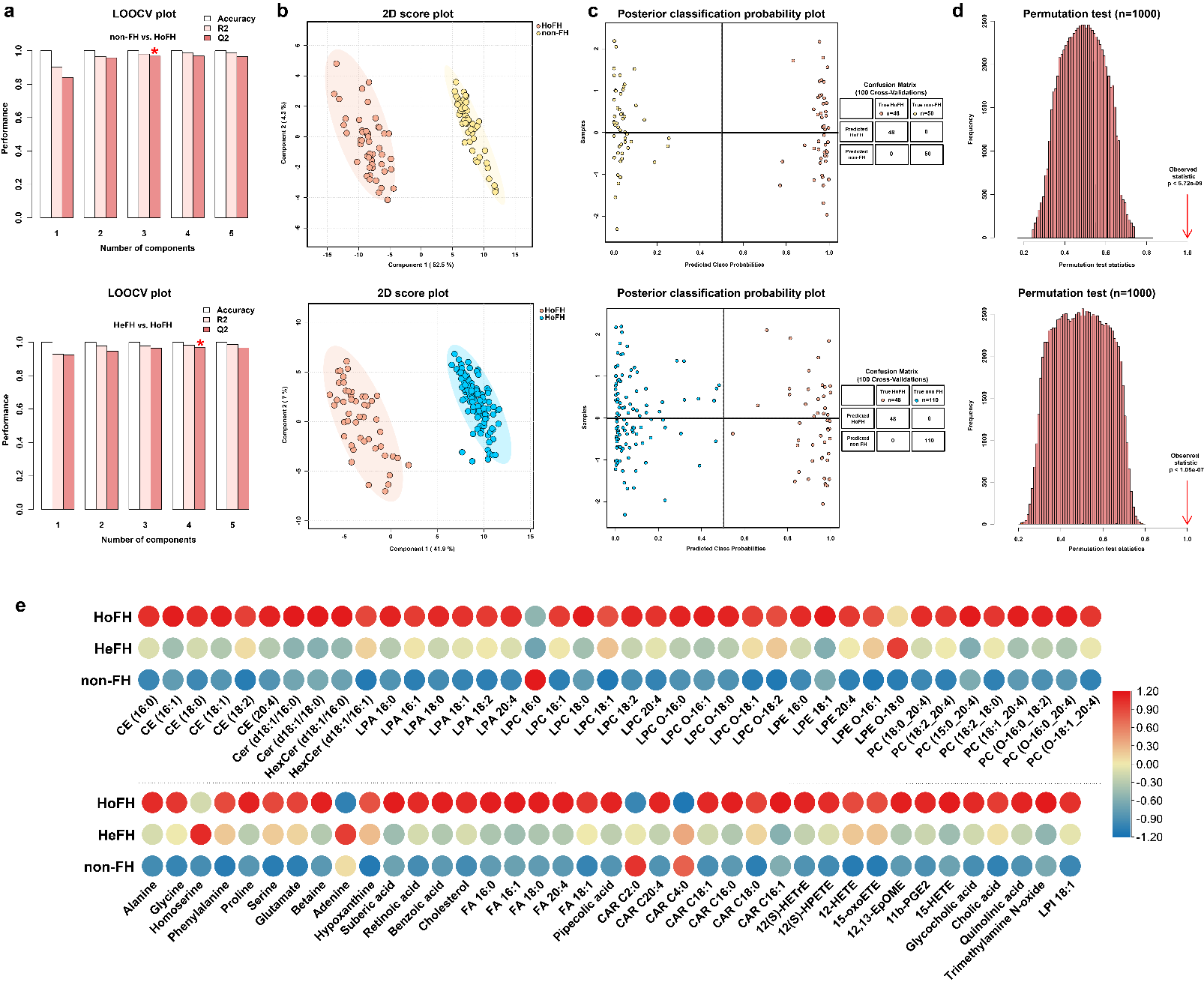


**Fig. S3** **PLS-DA model validation of the differentially expressed metabolites for discriminating HoFH from non-FH and HeFH in validation cohort.** **(a)** Performance measure plots of LOOCV. Q2 value > 0.4 in the first component was considered as a reliable model, the optimal numbers of component were labeled by a red star. **(b)** Two components-based PLS-DA scores plot of the metabolomic data; each point represents an individual serum sample. **(c)** Posterior classification probability plot based on 100 cross-validation. **(d)** Plots of permutation test based on 1000 times. **(e)** Heatmap of the differentiated metabolites that distinguished HoFH patients from HeFH patients and non-FH individuals in validation cohorts. FA = fatty acids, CE = cholesterol esters, Cer = ceramide, PC = diacylglycerophosphocholines, LPC = lyso PC, LPC-O = alkyl-LPC, LPE = lysophosphoethanolamines, LPE-O = alkyl-LPE, CAR = acyl carnitines, LPA = lysophosphatidic acid, HoFH = homozygous familial hypercholesterolemia; HeFH = heterozygous familial hypercholesterolemia; non-FH = non-familial hypercholesterolemia.


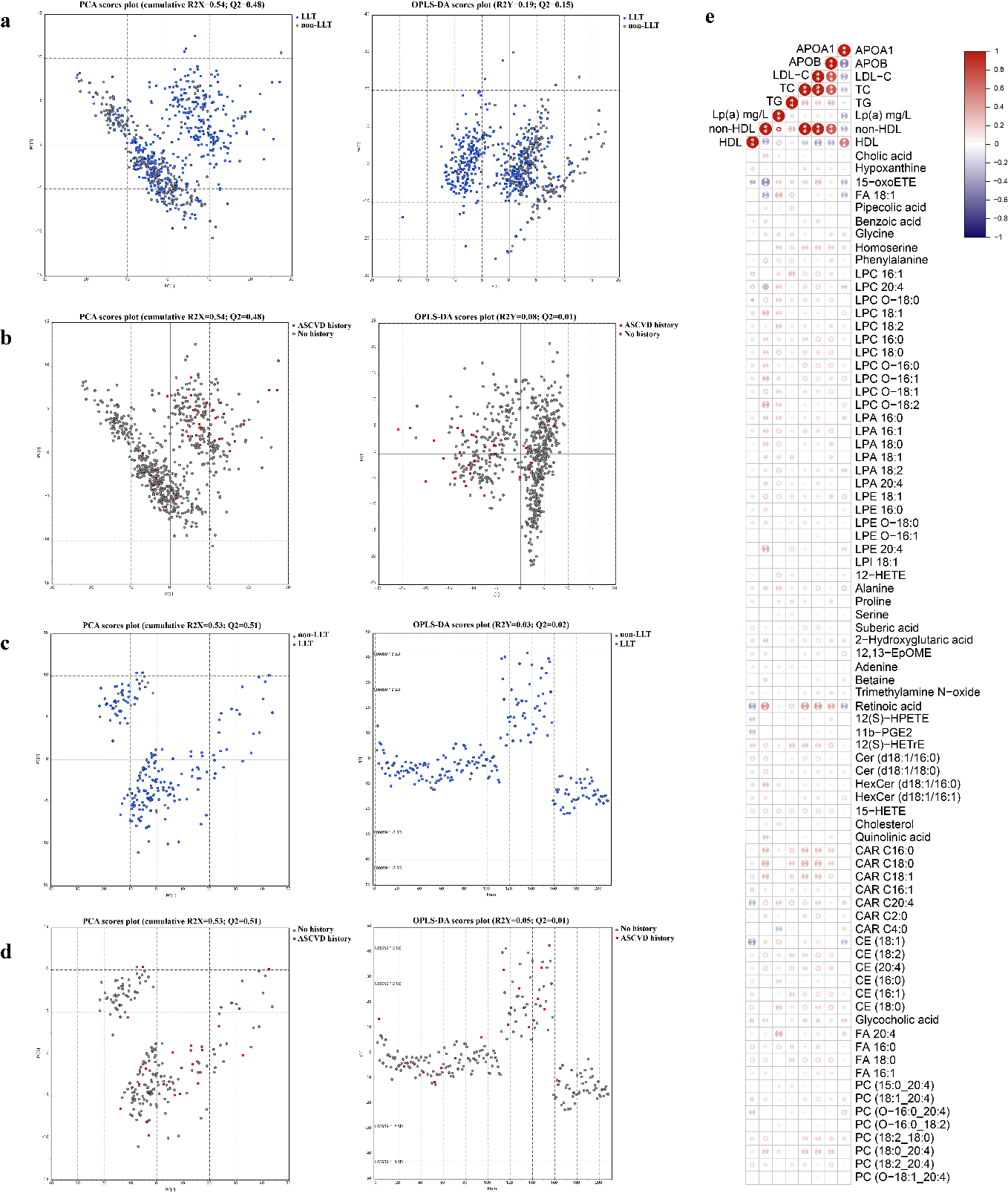


**Fig. S4 Correlation pattern analyses between discriminatory metabolites and clinical features.** **(a, b)** PCA and OPLS-DA score plots of discriminatory metabolite signatures from discovery cohort showing no variance due to LLT or CVD history. **(c, d)** PCA and OPLS-DA score plots of discriminatory metabolite signatures from validation cohort showing no variance due to LLT. All MVA analyses were based on pareto-scaled normalized datasets. **(e)** Spearman's rank correlation plot depicting the association of each differentiated metabolite with clinical lipid based on the merged datasets from discovery and validation cohorts. Positive correlations are displayed in red and negative correlations in blue, * = *P*<0.05, ** = *P*<0.01. Abbreviations are seen in Fig. S3.


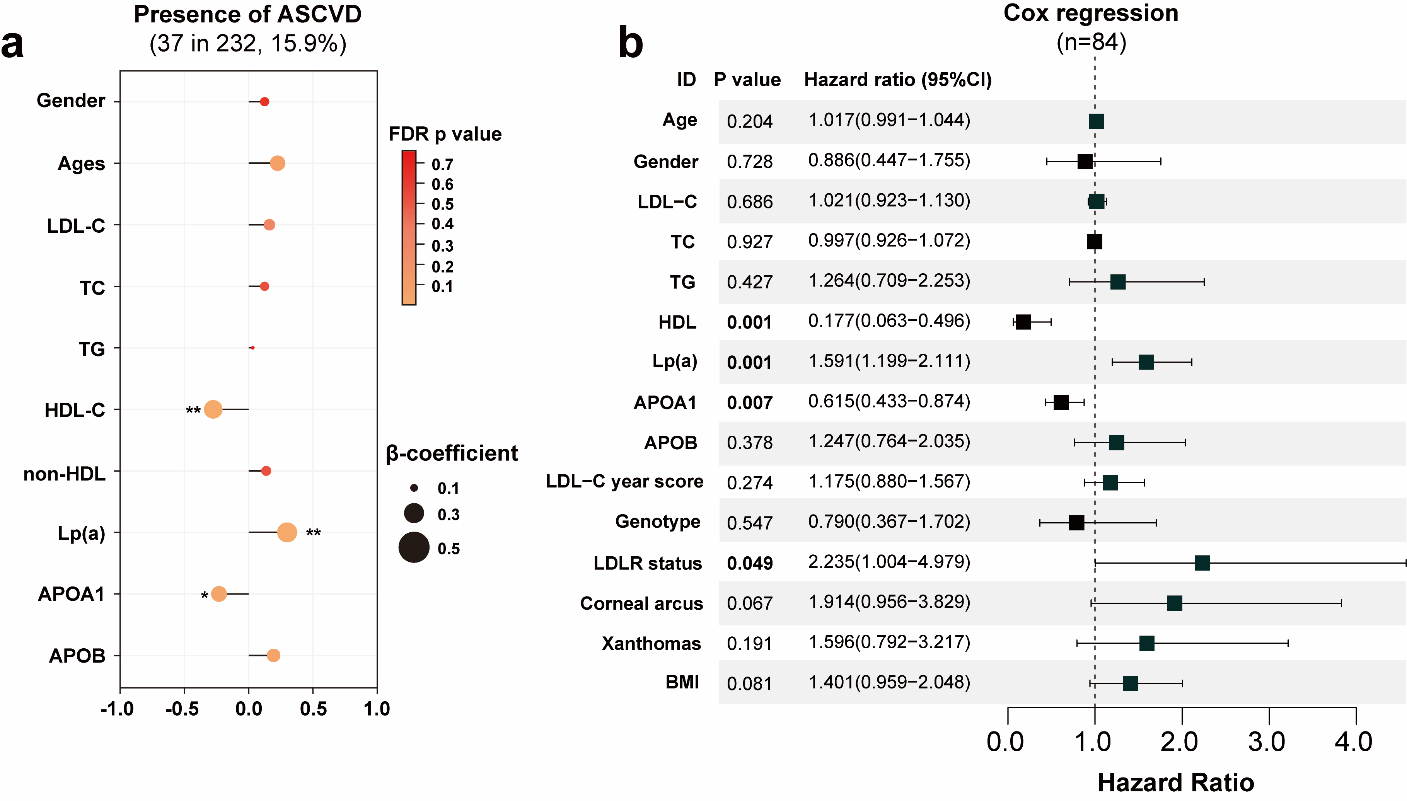


**Fig. S5** **The association of clinical/genetic risk factors with ASCVD.** **(a)** Correlation plot depicting the relation of clinical factors with the presence of ASCVD at baseline, using regression analyses (*P** < 0.05; *P*** < 0.01). In the 232 HoFH patients from discovery and validation cohorts, 37 patients had a ASCVD history. **(b)** Forest plot of unadjusted-hazard ratios and 95% confidence intervals (CI) depicting the association of clinical and genetical variables with incident ASCVD events during 4.9 years of follow up. LDL-C = low-density lipoprotein cholesterol, TC = total cholesterol, TG = triglycerides, HDL-C = high-density lipoprotein cholesterol, APOB = apolipoprotein B, APOA1 = apolipoprotein A1, Lp(a) = lipoprotein (a), BMI = body mass index.


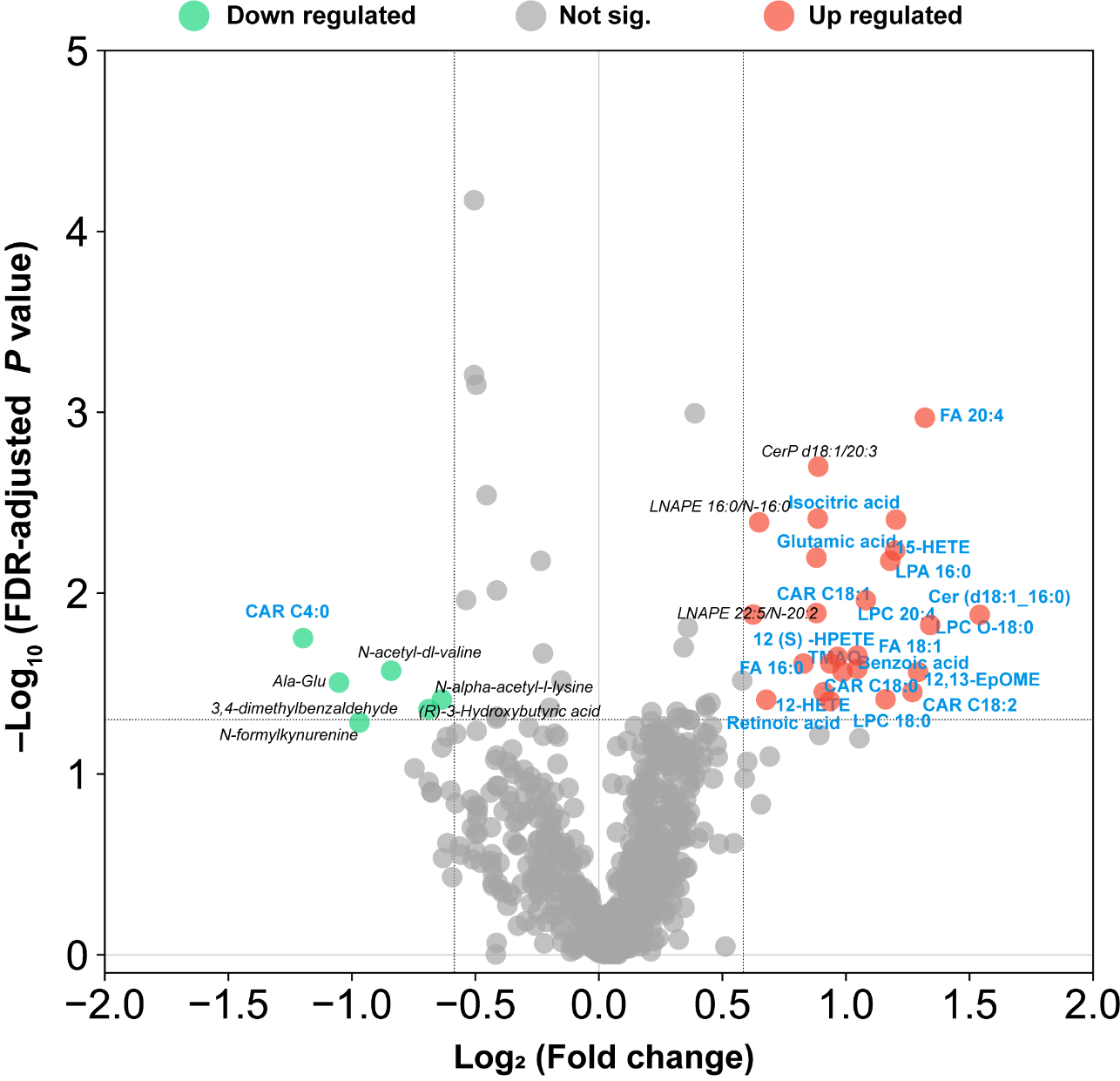


**Fig. S6** **Volcano plot of metabolite variations in pairwise comparisons of events vs. non-events.** in discovery and validation cohorts. The dashed lines indicate the threshold for the significant differences (FDR-adjusted *P* <0.05; Fold changes > 1.5). The chemical standards-annotated metabolites were labeled in blue color. FA = fatty acids, CE = cholesterol esters, Cer = ceramide, PC = diacylglycerophosphocholines, LPC = lyso PC, LPC-O = alkyl-LPC, CAR = acyl carnitines, LPA = lysophosphatidic acid, TMAO = trimethylamine-N-oxide.


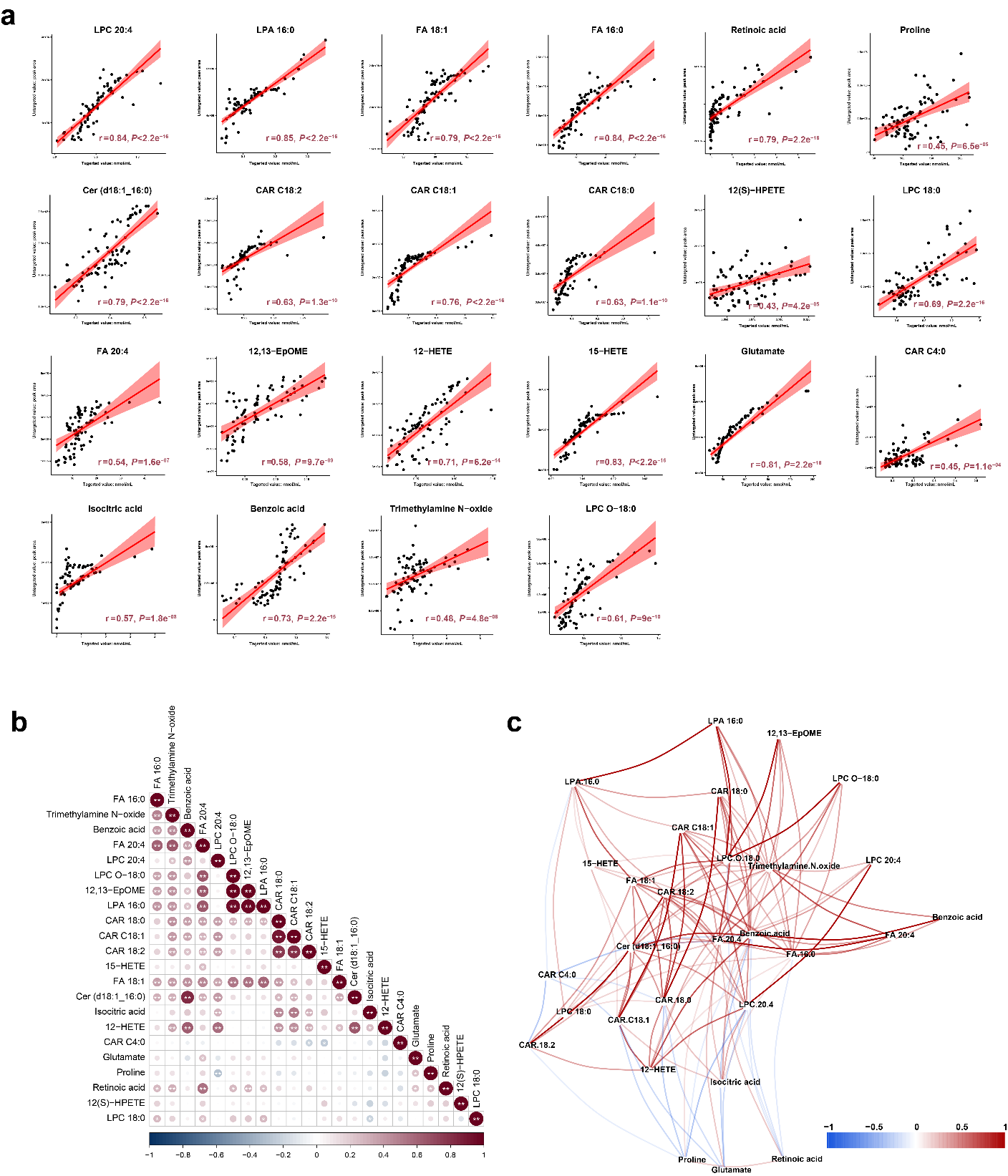


**Fig. S7** **Correlations between ASCVD-associated metabolomic markers.** **(a)** Pearson correlation analysis of the untargeted (semi-quantitative) and targeted (quantitative) measures values for sixteen ASCVD-related metabolites. **(b)** Spearman's rank correlation heat map based on the quantitative levels of sixteen metabolite; positive correlations are displayed in red and negative correlations in blue; color intensity and size of the circle are proportional to the Spearman correlation coefficients; correlations with *P* < 0.05* or *P* < 0.01** are considered significant. **(c)** Debiased sparse partial correlation network plot visualizing the major relationships of sixteen metabolite; circles represent metabolites, the thickness of the edges represents the strength of the partial correlation coefficients (coefficients ranges from 0.5 to 1.0). the red and blue line indicates positive and negative correlation, respectively. LPA 16:0 = lysophosphatidic acid 16:0, LPC-O 18:0 = lysophosphocholine alkyl−18:0, LPC 20:4 = lysophosphocholine 20:4, 12,13-EpOME = 12,13- epoxyoctadecenoic acid, Cer (d18:1_16:0) = Ceramide (d18:1_16:0), TMAO = trimethylamine-N-oxide. Other abbreviations are seen in Fig. S3.


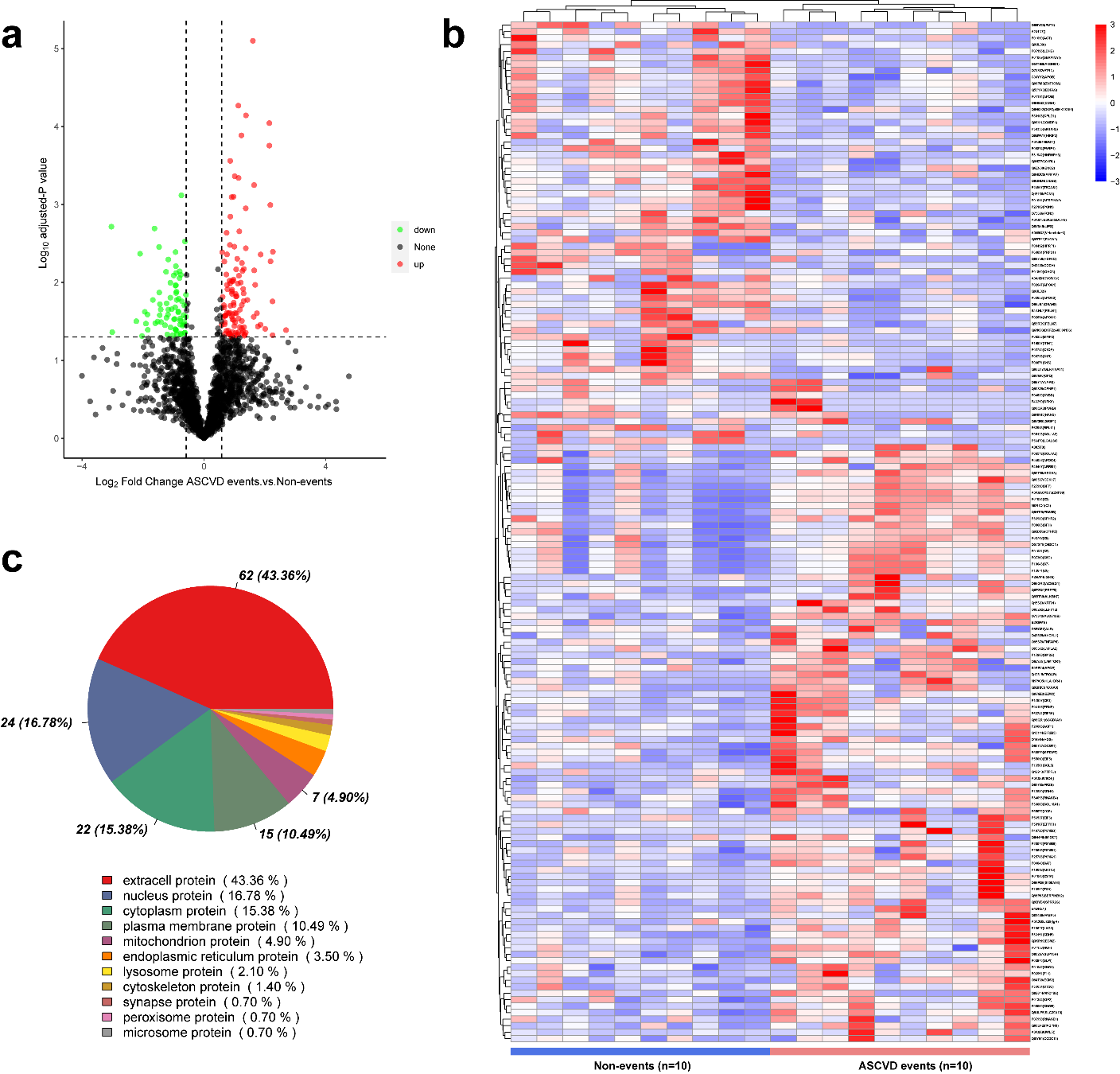


**Fig. S8** **Differentiated** **proteins identified in the comparison of patients with and without ASCVD events.** **(a)** Volcano plots compare two patient groups as indicated in the plot. Proteins with fold-change beyond 1.5 or below 0.67 with adjusted *P* value lower than 0.05 were considered as significantly differential expression. **(b)** Clustering heat map of the differentiated proteins that distinguished patients with ASCVD events from patients without events. **(c)** Distribution of subcellular localization of differential expressed proteins between the two study groups.

**Table S1 Demographic and clinical characteristics of all subjects in the validation cohort.**

|  | HoFH (n=48) | HeFH (n=110) | Non-FH (n=50) | *P* value |
| --- | --- | --- | --- | --- |
| Ages | 25.2±13.2 | 35.6±11.5 ^a^ | 27.9±7.6 | <0.0001 |
| Male sex, n (%) | 21 (43.8%) | 51 (46.4%) | 26 (52%) | 0.68 |
| Hypertension, n (%) | 6 (12.5%) | 13 (11.8%) | 9 (18%) | 0.56 |
| Diabetes mellitus, n (%) | 0 (0%) | 7 (6.4%) | 1 (2%) | 0.09 |
| Current smokers, n (%) | 0 (0%) | 8 (7.3%) | 3 (6%) | 0.14 |
| ASCVD history, n (%) | 12 (25.0%) | 12 (10.9%) ^a^ | 2 (4%) ^b^ | 0.0078 |
| Current corneal arcus, n (%) | 26 (54.2%) | 0 (0%) ^a^ | 0 (0%) ^b^ | <0.0001 |
| Current xanthomas, n (%) | 31 (64.6%) | 2 (1.8%) ^a^ | 0 (0%) ^b^ | <0.0001 |
| SVAS, n (%) | 9 (18.8%) | 0 (0.0%) ^a^ | 0 (0.0%) ^b^ | <0.0001 |
| Calcific VAS, n (%) | 13 (27.1%) | 0 (0.0%) ^a^ | 0 (0.0%) ^b^ | <0.0001 |
| LLT, n (%) | 48 (100%) | 95 (86.4%) ^a^ | 40 (80%) ^b^ | 0.0034 |
| LDL-C, mmol/L | 11.74±4.64 | 5.39±0.87 ^a^ | 2.33±0.51 ^b^ | <0.0001 |
| TC, mmol/L | 14.06±4.87 | 7.58±1.38 ^a^ | 4.29±1.09 ^b^ | <0.0001 |
| TG, mmol/L | 0.9 [0.7, 1.3] | 1.3 [0.94, 1.7] ^a^ | 1.1 [0.81, 1.53] | 0.0048 |
| HDL-C, mmol/L | 0.85±0.30 | 1.34±0.29 ^a^ | 1.37±0.34 ^b^ | <0.0001 |
| Non-HDL, mmol/L | 13.20±4.94 | 6.21±1.36 ^a^ | 2.95±1.10 ^b^ | <0.0001 |
| APOB, g/L | 2.41±0.60 | 1.51±0.22 ^a^ | 0.93±0.23 ^b^ | <0.0001 |
| APOA1, g/L | 0.88±0.31 | 1.08±0.29 ^a^ | 1.28±0.26 ^b^ | <0.0001 |
| LP(a), mg/dL | 39.8 [26.6, 64.8] | 15.5 [13.1, 18.8] ^a^ | 8.2 [5.9, 11.5] ^b^ | <0.0001 |

Continuous data are presented as mean ± standard deviation or median [interquartile range], and categorical variables are presented as %. ANOVA and consequent post hoc test were used for continuous data. The Chi-square test was used for categorical data. ^a^: *P* value <0.05 in the comparison of HeFH and HoFH; ^b^: *P* value <0.05 in the comparison of non-FH and HoFH; CVD, cardiovascular disease; LDL-C, low-density lipoprotein cholesterol; TC, total cholesterol; TG, triglycerides; HDL-C, high-density lipoprotein cholesterol; APOB, apolipoprotein B; APOA1, apolipoprotein A1; Lp(a), lipoprotein (a); SVAS, supravalvular aortic stenosis; VAS, valvular aortic stenosis; HoFH, homozygous familial hypercholesterolemia; HeFH, heterozygous familial hypercholesterolemia; Non-FH, non-familial hypercholesterolemia.

**Table S2** **Detailed information on** **lipid-lowering therapies in both cohorts.**

| Discovery cohort | | | |
| --- | --- | --- | --- |
| Lipid-lowering therapies (n) | HoFH (n=165) | HeFH (n=264) | non-FH (n=37) |
| Statin alone, n (%) | 15 (9.1%) | 185 (70.1%) | 31 (83.8%) |
| Statin + Probucol, n (%) | 16 (9.7%) | 7 (2.7%) | 0 (%) |
| Stain + Ezetimibe, n (%) | 100 (60.6%) | 54 (20.5%) | 6 (16.2%) |
| Stain + PCSK9 inhibitor, n (%) | 11 (6.7%) | 18 (6.8%) | 0 (%) |
| Stain + Ezetimibe + PCSK9 inhibitor, n (%) | 16 (9.7%) | 0 (0%) | 0 (%) |
| Lipoprotein apheresis, n (%) | 7 (4.2%) | 0 (0%) | 0 (%) |
| Validation cohort | | | |
| Lipid-lowering therapies (n) | HoFH (n=48) | HeFH (n=95) | Non-FH (n=40) |
| Statin alone, n (%) | 3 (6.3%) | 65 (68.4%) | 34 (85%) |
| Statin + Probucol, n (%) | 8 (16.7%) | 2 (2.1%) | 0 (0%) |
| Stain + Ezetimibe, n (%) | 23 (47.9%) | 21 (22.1%) | 6 (15%) |
| Stain + PCSK9 inhibitor, n (%) | 4 (8.3%) | 7 (7.4%) | 0 (0%) |
| Stain + Ezetimibe + PCSK9 inhibitor, n (%) | 8 (16.7%) | 0 (0%) | 0 (0%) |
| Lipoprotein apheresis, n (%) | 2 (4.2%) | 0 (0%) | 0 (0%) |

HoFH, homozygous familial hypercholesterolemia; HeFH, heterozygous familial hypercholesterolemia; Non-FH, non-familial hypercholesterolemia.

**Table S3.** **Metabolites for differentiating HoFH from HeFH and non-FH in the discovery cohort.**

|  | **HoFH vs. Non-FH** | | | **HoFH vs. Non-FH** | | |
| --- | --- | --- | --- | --- | --- | --- |
| Compound name | log2FC | P value | FDR | log2FC | P value | FDR |
| 11b-PGE2 | 1.24 | 8.88E-53 | 2.64E-51 | 0.88 | 6.96E-05 | 1.34E-04 |
| 12(S)-HPETE | 1.51 | 1.79E-19 | 3.88E-19 | 1.07 | 8.09E-03 | 1.15E-02 |
| 12,13-EpOME | 1.82 | 1.79E-19 | 3.81E-19 | 1.03 | 8.09E-03 | 1.13E-02 |
| 12-HETE | 1.15 | 1.63E-25 | 5.38E-25 | 0.67 | 1.58E-10 | 4.47E-10 |
| 15-HETE | 1.40 | 7.64E-43 | 1.14E-41 | 1.13 | 3.23E-05 | 6.40E-05 |
| 15-oxoETE | 1.80 | 3.12E-42 | 4.13E-41 | 1.23 | 1.06E-08 | 2.64E-08 |
| 2-Hydroxyglutaric acid | 1.51 | 1.36E-20 | 3.11E-20 | 0.34 | 5.01E-02 | 5.96E-02 |
| 3-Nitrotyrosine | 1.33 | 1.06E-25 | 3.70E-25 | 1.29 | 4.05E-01 | 4.42E-01 |
| Adenine | -0.64 | 3.34E-12 | 5.16E-12 | -1.10 | 3.66E-33 | 8.72E-32 |
| Alanine | 1.13 | 2.77E-38 | 2.75E-37 | 0.68 | 1.83E-25 | 1.81E-24 |
| Aspartate | 1.44 | 1.62E-16 | 2.89E-16 | 0.79 | 8.68E-01 | 8.83E-01 |
| Benzoic acid | 1.30 | 5.48E-57 | 3.26E-55 | 0.70 | 1.25E-52 | 4.96E-51 |
| Betaine | 1.01 | 3.07E-15 | 5.15E-15 | 0.77 | 4.95E-09 | 1.31E-08 |
| CAR C16:0 | 0.94 | 3.15E-18 | 6.15E-18 | 0.72 | 1.05E-18 | 6.23E-18 |
| CAR C16:1 | 1.24 | 5.23E-07 | 7.24E-07 | 0.60 | 2.93E-11 | 8.95E-11 |
| CAR C18:0 | 1.36 | 5.41E-22 | 1.34E-21 | 1.11 | 2.85E-18 | 1.48E-17 |
| CAR C18:1 | 1.21 | 9.29E-14 | 1.49E-13 | 0.57 | 2.53E-18 | 1.37E-17 |
| CAR C18:2 | 1.19 | 2.39E-16 | 4.13E-16 | 0.44 | 1.18E-01 | 1.34E-01 |
| CAR C2:0 | -1.99 | 3.86E-55 | 1.53E-53 | -0.96 | 2.35E-08 | 5.71E-08 |
| CAR C20:4 | 2.11 | 2.01E-49 | 3.99E-48 | 1.36 | 1.19E-36 | 3.54E-35 |
| CAR C4:0 | -1.64 | 1.19E-26 | 4.59E-26 | -0.88 | 7.33E-25 | 6.23E-24 |
| CE (16:0) | 1.72 | 3.44E-06 | 4.66E-06 | 1.50 | 2.90E-04 | 5.16E-04 |
| CE (16:1) | 1.20 | 6.94E-03 | 7.72E-03 | 0.67 | 3.16E-02 | 3.80E-02 |
| CE (18:0) | 1.79 | 1.94E-03 | 2.36E-03 | 1.18 | 2.71E-02 | 3.43E-02 |
| CE (18:1) | 1.58 | 1.69E-03 | 2.10E-03 | 0.95 | 1.27E-02 | 1.73E-02 |
| CE (18:2) | 2.44 | 3.09E-04 | 4.04E-04 | 1.43 | 3.55E-03 | 5.48E-03 |
| CE (20:4) | 1.52 | 1.23E-04 | 1.62E-04 | 0.85 | 4.24E-04 | 7.41E-04 |
| Cer (d18:1_16:0) | 1.38 | 7.40E-04 | 9.57E-04 | 0.78 | 4.61E-03 | 6.86E-03 |
| Cer (d18:1_18:0) | 1.52 | 2.94E-03 | 3.46E-03 | 1.07 | 2.10E-02 | 2.78E-02 |
| Chenodeoxycholic acid | 1.56 | 1.31E-02 | 1.40E-02 | 1.07 | 5.12E-01 | 5.54E-01 |
| Cholesterol | 1.69 | 3.03E-03 | 3.54E-03 | 0.94 | 4.27E-04 | 7.36E-04 |
| Cholic acid | 0.88 | 1.14E-03 | 1.45E-03 | 0.66 | 1.61E-02 | 1.81E-02 |
| Citrulline | 0.63 | 8.13E-12 | 1.24E-11 | -1.47 | 1.66E-05 | 3.46E-05 |
| FA 16:0 | 1.42 | 2.94E-27 | 1.20E-26 | 1.07 | 3.97E-32 | 6.76E-31 |
| FA 16:1 | 1.62 | 1.74E-03 | 2.14E-03 | 1.31 | 2.60E-02 | 3.77E-02 |
| FA 18:0 | 0.91 | 1.09E-51 | 2.58E-50 | 0.61 | 2.85E-02 | 3.14E-02 |
| FA 20:4 | 1.72 | 5.73E-21 | 1.34E-20 | 1.10 | 2.15E-25 | 1.96E-24 |
| FA18:1 | 1.20 | 5.10E-67 | 6.07E-65 | 0.82 | 4.44E-26 | 4.80E-25 |
| FA18:2 | 1.94 | 6.69E-16 | 1.14E-15 | 1.19 | 1.18E-01 | 1.35E-01 |
| Glutamate | 0.92 | 1.85E-18 | 3.72E-18 | 0.62 | 2.92E-03 | 4.69E-03 |
| Glycine | 1.17 | 5.58E-23 | 1.48E-22 | 0.74 | 1.61E-12 | 5.62E-12 |
| Glycocholic acid | 1.45 | 1.47E-03 | 1.86E-03 | 0.88 | 7.06E-03 | 1.02E-02 |
| Glycoursodeoxycholic acid | 2.43 | 4.27E-03 | 4.79E-03 | 0.94 | 8.62E-01 | 8.84E-01 |
| HexCer (d18:1_16:0) | 2.11 | 3.86E-03 | 4.41E-03 | 1.04 | 2.85E-02 | 3.54E-02 |
| HexCer (d18:1_16:1) | 2.12 | 1.52E-03 | 1.90E-03 | 0.75 | 6.76E-03 | 9.94E-03 |
| Histidine | 1.50 | 7.99E-09 | 1.15E-08 | -1.36 | 2.42E-10 | 6.70E-10 |
| Homoserine | 1.19 | 4.40E-33 | 3.27E-32 | -1.07 | 1.10E-02 | 1.53E-02 |
| Hypoxanthine | 1.30 | 1.62E-17 | 3.02E-17 | 0.90 | 1.20E-02 | 1.68E-02 |
| Indole-3-carboxylic acid | 1.08 | 1.28E-10 | 1.91E-10 | 1.63 | 2.84E-03 | 4.63E-03 |
| Isocitric acid | 1.30 | 7.55E-13 | 1.20E-12 | 0.57 | 1.46E-14 | 8.70E-13 |
| Isoleucine | 1.23 | 5.87E-19 | 1.20E-18 | -0.65 | 6.19E-01 | 6.52E-01 |
| LPA 16:0 | 1.80 | 1.05E-29 | 5.94E-29 | 0.99 | 3.29E-08 | 7.68E-08 |
| LPA 16:1 | 0.96 | 6.23E-32 | 4.12E-31 | 0.88 | 2.86E-19 | 1.89E-18 |
| LPA 18:0 | 1.04 | 2.93E-18 | 5.80E-18 | 1.33 | 7.47E-13 | 2.78E-12 |
| LPA 18:1 | 1.66 | 8.53E-15 | 1.41E-14 | 1.31 | 1.11E-10 | 3.22E-10 |
| LPA 18:2 | 1.95 | 1.96E-12 | 3.08E-12 | 1.75 | 1.88E-07 | 4.29E-07 |
| LPA 20:4 | 2.52 | 1.26E-21 | 3.00E-21 | 1.38 | 2.53E-08 | 6.03E-08 |
| LPC 16:0 | 2.04 | 1.22E-28 | 6.03E-28 | 1.72 | 3.98E-15 | 1.53E-14 |
| LPC 16:1 | 2.12 | 2.31E-28 | 1.06E-27 | 1.38 | 1.77E-05 | 3.63E-05 |
| LPC 18:0 | 1.87 | 6.23E-32 | 3.90E-31 | 1.16 | 2.86E-19 | 1.79E-18 |
| LPC 18:1 | 1.04 | 3.86E-25 | 1.24E-24 | 1.04 | 1.17E-04 | 2.18E-04 |
| LPC 18:2 | 1.54 | 8.00E-26 | 2.89E-25 | 0.90 | 3.25E-05 | 6.34E-05 |
| LPC 20:4 | 2.06 | 1.01E-39 | 1.21E-38 | 0.98 | 2.74E-30 | 4.08E-29 |
| LPC O-16:0 | 2.05 | 1.29E-27 | 5.67E-27 | 1.31 | 3.67E-12 | 1.18E-11 |
| LPC O-16:1 | 1.95 | 7.44E-36 | 6.81E-35 | 1.76 | 5.71E-09 | 1.48E-08 |
| LPC O-18:0 | 2.10 | 9.10E-33 | 6.37E-32 | 1.76 | 1.12E-18 | 6.36E-18 |
| LPC O-18:1 | 2.07 | 2.25E-33 | 1.79E-32 | 1.35 | 1.10E-17 | 5.02E-17 |
| LPC O-18:2 | 1.74 | 1.88E-27 | 8.00E-27 | 1.20 | 8.54E-12 | 2.67E-11 |
| LPE 16:0 | 1.69 | 3.14E-27 | 1.24E-26 | 1.05 | 2.33E-17 | 1.11E-16 |
| LPE 18:0 | 1.48 | 3.54E-06 | 4.74E-06 | -0.88 | 2.36E-12 | 8.01E-12 |
| LPE 18:1 | 1.39 | 4.42E-25 | 1.39E-24 | 0.81 | 1.26E-06 | 2.82E-06 |
| LPE 18:2 | 2.13 | 1.48E-10 | 2.17E-10 | -0.87 | 3.14E-02 | 3.82E-02 |
| LPE 20:4 | 1.95 | 1.52E-25 | 5.17E-25 | 1.54 | 2.66E-06 | 5.75E-06 |
| LPE O-16:1 | 2.09 | 1.36E-09 | 1.98E-09 | 0.70 | 7.81E-05 | 1.48E-04 |
| LPE O-18:0 | 1.10 | 3.65E-03 | 4.21E-03 | 0.85 | 2.77E-02 | 3.47E-02 |
| LPI 18:1 | 1.24 | 6.17E-24 | 1.71E-23 | 0.69 | 1.85E-06 | 4.07E-06 |
| LPI 20:4 | 1.52 | 2.26E-07 | 3.16E-07 | 0.78 | 7.19E-01 | 7.51E-01 |
| Ornithine | 0.66 | 7.30E-24 | 1.98E-23 | -1.26 | 5.47E-06 | 1.16E-05 |
| PC (15:0_20:4) | 2.21 | 1.00E-28 | 5.18E-28 | 1.73 | 6.43E-55 | 7.66E-53 |
| PC (18:0_20:4) | 2.41 | 1.49E-06 | 2.03E-06 | 1.04 | 2.68E-16 | 1.14E-15 |
| PC (18:1_20:4) | 0.99 | 5.13E-25 | 1.57E-24 | 0.64 | 8.62E-09 | 2.18E-08 |
| PC (18:2_18:0) | 1.96 | 1.84E-28 | 8.76E-28 | 1.20 | 1.46E-03 | 2.48E-03 |
| PC (18:2_20:4) | 2.06 | 3.11E-19 | 6.49E-19 | 1.32 | 1.70E-03 | 2.81E-03 |
| PC (O-16:0_18:2) | 1.53 | 1.89E-17 | 3.47E-17 | 0.97 | 4.70E-02 | 4.71E-02 |
| PC (O-16:0_20:4) | 1.28 | 6.16E-08 | 8.72E-08 | 1.20 | 1.18E-16 | 5.20E-16 |
| PC (O-18:1_20:4) | 1.15 | 1.05E-11 | 1.58E-11 | 0.84 | 3.16E-16 | 1.30E-15 |
| Phenylalanine | 1.18 | 7.17E-22 | 1.74E-21 | 0.89 | 9.68E-03 | 9.76E-02 |
| Pipecolic acid | 0.82 | 9.73E-39 | 1.05E-37 | 0.88 | 3.40E-21 | 2.38E-20 |
| Proline | 1.65 | 1.32E-29 | 7.16E-29 | 0.80 | 2.73E-12 | 9.02E-12 |
| Pyroglutamic acid | 0.96 | 1.23E-45 | 2.08E-44 | -0.41 | 7.27E-03 | 1.04E-02 |
| Quinolinic acid | 2.12 | 3.86E-03 | 4.37E-03 | 0.81 | 2.85E-02 | 3.50E-02 |
| Retinoic acid | 0.93 | 2.36E-14 | 3.85E-14 | 0.97 | 5.02E-33 | 9.95E-32 |
| Serine | 1.89 | 8.24E-23 | 2.13E-22 | 1.41 | 5.72E-11 | 1.70E-10 |
| Suberic acid | 1.18 | 1.52E-26 | 5.64E-26 | 0.74 | 5.79E-16 | 2.30E-15 |
| Threonine | 0.81 | 7.63E-18 | 1.44E-17 | -0.38 | 5.99E-01 | 6.42E-01 |
| Trimethylamine N-oxide | 1.20 | 3.44E-18 | 6.59E-18 | 0.69 | 2.47E-02 | 3.19E-02 |

Two-tailed Student’s *t* test or Mann Whitney *U* test were used for each comparison. FC= fold change. FA = fatty acids, CE = cholesterol esters, Cer = ceramide, CerP = Cer-1-phosphate, HexCer = Hexosyl-Cer, LPA = lysophosphatidic acid, CAR = acyl carnitines, PC = diacylglycerophosphocholines, PC-O = alkyl-PC, LPC = lyso PC, LPC-O = alkyl-LPC, LPE = lysophosphoethanolamines, LPE-O = alkyl-LPE, TG = triglycerides, LPI = lysophosphatidylinositol, HoFH, homozygous familial hypercholesterolemia; HeFH, heterozygous familial hypercholesterolemia; Non-FH, non-familial hypercholesterolemia.

**Table S4. Associations between serum metabolites and corneal arcus/xanthomas by using regression analyses.**

|  | Univariate regression | | Multivariate regression | |
| --- | --- | --- | --- | --- |
| Metabolites | β-coefficient | FDR-*P* value | β-coefficient | FDR-*P* value |
| FA 18:2 | 0.509 | 0.001 | 0.384 | 0.029 |
| FA 18:1 | 0.37 | 0.017 | 0.249 | 0.14 |
| FA 16:0 | 0.412 | 0.01 | 0.352 | 0.033 |
| LPC 20:4 | 0.83 | <0.0001 | 0.653 | 0.001 |
| PC (18:2_20:4) | 0.322 | 0.036 | 0.2 | 0.238 |
| LPA 18:1 | 0.33 | 0.042 | 0.328 | 0.044 |
| LPA 18:2 | 0.326 | 0.041 | 0.243 | 0.15 |
| LPA 20:4 | 0.501 | 0.003 | 0.38 | 0.032 |
| CAR C2:0 | -0.407 | 0.025 | -0.402 | 0.042 |
| CAR C4:0 | -0.582 | 0.006 | -0.447 | 0.041 |
| Taurocholic acid | 0.534 | 0.001 | 0.497 | 0.004 |
| 12(S)-HPETE | 0.364 | 0.022 | 0.226 | 0.201 |
| 11b-PGE2 | 0.515 | 0.001 | 0.449 | 0.009 |
| 15-oxoETE | 0.651 | <0.0001 | 0.583 | 0.002 |
| 12-HETE | 0.331 | 0.034 | 0.186 | 0.252 |

Adjusted-covariates in multivariate regression including age, sex, and clinical lipids. β-coefficient >0 or <0 combined with a FDR-adjusted *P* value <0.05 indicated a positive or negative correlation with corneal arcus/xanthomas. FA = fatty acid, CAR = acyl carnitines, 12-HETE = 12-hydroxy-eicosatetraenoic acid; LPA = lysophosphatidic acid, PC (18:2_20:4) = diacylglycerophosphocholines 18:2_20:4, LPC 20:3 = lysophosphocholine 20:3, 11b-PGE2 = 11β-prostaglandin E2, 12(S)-HPETE = 12(S)-hydroperoxyeicosatetraenoic acid, 15-oxoETE, 15-oxo-eicosatetraenoate.

**Table S5. Associations between serum metabolites and supravalvular aortic stenosis by using regression analyses.**

|  | Univariate regression | | Multivariate regression | |
| --- | --- | --- | --- | --- |
| Metabolites | β-coefficient | FDR-*P* value | β-coefficient | FDR-*P* value |
| LPC 20:4 | 1.048 | <0.0001 | 0.948 | <0.0001 |
| Alanine | 0.586 | 0.001 | 0.455 | 0.032 |
| Benzoic acid | 0.521 | 0.01 | 0.441 | 0.049 |
| 11b-PGE2 | 0.498 | 0.004 | 0.374 | 0.085 |
| LPC 18:0 | 0.315 | 0.013 | 0.4 | 0.043 |
| LPE O-16:1 | 0.544 | 0.001 | 0.566 | 0.003 |
| CE (16:0) | 0.291 | 0.038 | 0.108 | 0.585 |
| Retinoic acid | 0.536 | 0.002 | 0.572 | 0.01 |
| LPA 20:4 | 0.46 | 0.008 | 0.378 | 0.042 |
| LPE O-18:0 | 0.464 | 0.004 | 0.709 | 0.001 |
| LPI 18:1 | 0.359 | 0.046 | 0.856 | 0.002 |
| CE (18:0) | 0.445 | 0.007 | 0.226 | 0.28 |

Adjusted-covariates in multivariate regression including sex, age, and clinical lipids. β-coefficient >0 or <0 combined with a FDR-adjusted *P* value <0.05 indicated a positive or negative correlation with corneal arcus/xanthomas. 20-HETE = 20-hydroxy-eicosatetraenoic acid; LPA = lysophosphatidic acid, LPC = lysophosphocholine, 11b-PGE2 = 11β-prostaglandin E2, LPE = lysophosphoethanolamines, LPI = lysophosphatidylinositol, 15-oxoETE, 15-oxo-eicosatetraenoate.

**Table S6. Associations between serum metabolites and calcific valvular aortic stenosis by using regression analyses.**

|  | Univariate regression | | Multivariate regression | |
| --- | --- | --- | --- | --- |
| Metabolites | β-coefficient | FDR-*P* value | β-coefficient | FDR-*P* value |
| LPA 18:0 | 1.435 | <0.0001 | 1.442 | <0.0001 |
| LPC 20:4 | 0.313 | 0.039 | 0.088 | 0.701 |
| Retinoic acid | 0.801 | <0.0001 | 1.106 | <0.0001 |
| LPA 18:2 | 0.62 | <0.0001 | 0.686 | 0.002 |
| LPA 16:0 | 0.166 | 0.3 | 0.203 | 0.299 |
| Alanine | 0.371 | 0.023 | 0.401 | 0.045 |
| LPA 20:4 | 0.429 | 0.014 | 0.33 | 0.101 |
| CAR C18:1 | 0.371 | 0.023 | 0.401 | 0.045 |
| 11b-PGE2 | 0.558 | 0.002 | 0.65 | 0.003 |
| FA 20:4 | 0.321 | 0.058 | 0.378 | 0.055 |
| 15-HETE | 0.218 | 0.213 | 0.376 | 0.031 |
| Proline | 0.48 | 0.006 | 0.576 | 0.011 |

Adjusted-covariates in multivariate regression including age, sex, and clinical lipids. β-coefficient >0 or <0 combined with a FDR-adjusted *P* value <0.05 indicated a positive or negative correlation with corneal arcus/xanthomas. 15-HETE = 15-hydroxy-eicosatetraenoic acid; LPA = lysophosphatidic acid, LPC = lysophosphocholine, 11b-PGE2 = 11β-prostaglandin E2.

**Table S7. Associations between serum metabolites and the presence of ASCVD by using regression analyses.**

|  | Univariate regression | | Multivariate regression | |
| --- | --- | --- | --- | --- |
| Metabolites | β-coefficient | FDR-*P* value | β-coefficient | FDR-*P* value |
| 12,13-EpOME | 0.354 | <0.0001 | 0.312 | <0.0001 |
| 12-HETE | 0.321 | 0.0002 | 0.286 | 0.0062 |
| 15-HETE | 0.256 | 0.0045 | 0.251 | 0.0051 |
| 15-oxoETE | 0.302 | 0.011 | 0.31 | 0.034 |
| CAR C16:0 | 0.183 | 0.0014 | 0.145 | 0.042 |
| CAR C18:0 | 0.232 | 0.042 | 0.178 | 0.024 |
| Cer (d18:1_16:0) | 0.456 | <0.0001 | 0.422 | <0.0001 |
| FA 16:0 | 0.166 | 0.022 | 0.134 | 0.043 |
| FA 18:0 | 0.175 | 0.008 | 0.139 | 0.088 |
| FA 20:4 | 0.294 | 0.0023 | 0.282 | 0.0029 |
| LPA 16:0 | 0.427 | <0.0001 | 0.387 | <0.0001 |
| LPA 18:0 | 0.325 | <0.0001 | 0.304 | <0.0001 |
| LPC 18:0 | 0.283 | 0.0075 | 0.263 | 0.015 |
| LPC 20:4 | 0.39 | <0.0001 | 0.364 | <0.0001 |

In the 232 HoFH patients from discovery and validation cohorts, 37 patients had a ASCVD history. Adjusted-covariates in multivariate regression including sex, age, and clinical lipids. β-coefficient >0 or <0 combined with a FDR-adjusted *P* value <0.05 indicated a positive or negative correlation with corneal arcus/xanthomas. 15-HETE = 15-hydroxy-eicosatetraenoic acid; LPA = lysophosphatidic acid, LPC = lysophosphocholine, 12,13-EpOME = 12,13- epoxyoctadecenoic acid, Cer (d18:1_16:0) = Ceramide (d18:1_16:0), CAR = acyl carnitines, FA = fatty acid.

**Table S8.** **Differentiated metabolites identified in the comparison of HoFH patients with and without ASCVD events.**

| **Metabolite** | **Classes** | **Metabolic pathways** | **Events vs. No events: Log2 (FC)** | **FDR *P* value** | **CV** |
| --- | --- | --- | --- | --- | --- |
| 12(S)-HPETE | Oxylipins | Arachidonic acid metabolism | 0.958 | 2.19E-02 | 0.15 |
| 12,13-EpOME | Oxylipins | Arachidonic acid metabolism | 1.362 | 2.39E-02 | 0.14 |
| 12-HETE | Oxylipins | Arachidonic acid metabolism | 0.956 | 3.77E-02 | 0.12 |
| 15-HETE | Oxylipins | Arachidonic acid metabolism | 1.230 | 6.25E-03 | 0.11 |
| Benzoic acid | Organic acids | Lipid transport and metabolism | 1.083 | 2.81E-02 | 0.14 |
| CAR C18:0 | Acylcarnitines | Carnitine synthesis | 1.133 | 3.07E-02 | 0.12 |
| CAR C18:1 | Acylcarnitines | Carnitine synthesis | 1.086 | 1.54E-02 | 0.15 |
| CAR C18:2 | Acylcarnitines | Carnitine synthesis | 1.281 | 3.32E-02 | 0.13 |
| CAR C4:0 | Acylcarnitines | Carnitine synthesis | -1.218 | 1.68E-02 | 0.1 |
| Cer (d18:1_16:0) | Ceramides | Sphingolipid metabolism | 1.531 | 1.32E-02 | 0.17 |
| FA 16:0 | Fatty acids | Fatty acid biosynthesis | 0.882 | 2.10E-02 | 0.085 |
| FA 18:1 | Fatty acids | Fatty acid biosynthesis | 1.163 | 2.41E-02 | 0.11 |
| FA 20:4 | Fatty acids | Arachidonic acid metabolism | 1.362 | 1.22E-03 | 0.043 |
| Glutamic acid | Amino acids | Glutamate metabolism | 0.956 | 6.84E-03 | 0.12 |
| Isocitric acid | Organic acids | TCA cycle | 0.911 | 4.03E-03 | 0.094 |
| LPA 16:0 | Lysophospholipids | Phospholipid biosynthesis | 1.208 | 6.33E-03 | 0.12 |
| LPC 18:0 | Lysophospholipids | Phospholipid biosynthesis | 1.289 | 3.43E-02 | 0.13 |
| LPC 20:4 | Lysophospholipids | Phospholipid biosynthesis | 1.165 | 1.11E-02 | 0.11 |
| LPC O-18:0 | Lysophospholipids | Phospholipid biosynthesis | 1.445 | 1.63E-02 | 0.15 |
| Proline | Amino acids | Arginine and proline metabolism | 0.880 | 5.00E-02 | 0.082 |
| Retinoic acid | Retinoids | Retinol metabolism | 0.832 | 5.00E-02 | 0.19 |
| Trimethylamine N-oxide | Amines | Betaine metabolism | 0.891 | 2.31E-02 | 0.18 |

*P* values were calculated by Mann Whitney *U* test and corrected for false discovery rate (FDR). LPA 16:0 = lysophosphatidic acid 16:0, LPC-O 18:0 = lysophosphocholine alkyl−18:0, LPC 20:4 = lysophosphocholine 20:4, 12,13-EpOME = 12,13- epoxyoctadecenoic acid, Cer (d18:1_16:0) = Ceramide (d18:1_16:0), HETE = hydroxy-eicosatetraenoic acid, CAR C= acyl carnitines, FA = fatty acids, HETE = hydroxy-eicosatetraenoic acid. CV = coefficients of variation in targeted analysis.

**Table S9.** **Concentrations of ASCVD-associated metabolites in HoFH patients with at least one LDLR mutation.**

| **ID** | **No event subjects (n=27)** | **Event subjects (n=25)** | **P value** |
| --- | --- | --- | --- |
| Null/Null mutations, n(%) | 6 (22.2%) | 11 (44.0%) | 0.094 |
| Null/Defective mutations, n(%) | 21 (77.8%) | 14 (56%) | 0.094 |
| FA 16:0 | 17.29 [11.78, 24.02] | 28.93 [19.44, 41.77] | 0.035 |
| FA 20:4 | 10.84 [7.84, 12.67] | 14.67 [12.64, 20.01] | <0.0001 |
| FA 18:1 | 49.79 [42.18, 57.27] | 62.79 [50.63, 73.07] | 0.0055 |
| LPA 16:0 | 0.087 [0.064, 0.12] | 0.13 [0.093, 0.19] | 0.0044 |
| LPC 20:4 | 0.43 [0.29, 0.55] | 0.57 [0.43, 0.77] | 0.0058 |
| LPC O-18:0 | 0.36 [0.26, 0.48] | 0.48 [0.42, 0.69] | 0.019 |
| 12,13-EpOME | 0.044 [0.032, 0.064] | 0.06 [0.047, 0.086] | 0.042 |
| 12-HETE | 0.048 [0.033, 0.059] | 0.061 [0.049, 0.094] | 0.037 |
| 15-HETE | 0.0071 [0.0045, 0.010] | 0.011 [0.0076, 0.014] | 0.0065 |
| CAR C18:0 | 0.077 [0.058, 0.11] | 0.09 [0.078, 0.13] | 0.0091 |
| CAR C18:1 | 0.15 [0.11, 0.24] | 0.27 [0.20, 0.31] | 0.0003 |
| CAR C18:2 | 0.22 [0.18, 0.26] | 0.28 [0.22, 0.33] | 0.018 |
| Cer (d18:1_16:0) | 0.33 [0.24, 0.43] | 0.44 [0.36, 0.49] | 0.014 |
| TMAO | 1.54 [1.11, 2.26] | 2.37 [1.69, 2.75] | 0.0065 |
| Isocitric acid | 0.54 [0.19, 0.90] | 0.67 [0.41, 1.10] | 0.12 |
| Benzoic acid | 0.95 [0.71, 1.002] | 1.03 [0.86, 1.18] | 0.11 |
| Glutamate | 51.2 [40.56, 65.46] | 53,02 [44.15, 94.15] | 0.15 |
| Proline | 109.94 [89.32, 131.33] | 106.84 [94.07, 137.95] | 0.94 |
| Retinoic acid | 0.2 [0.065, 0.58] | 0.37 [0.075, 1.14] | 0.36 |
| 12(S)-HPETE | 0.063 [0.05, 0.082] | 0.068 [0.053, 0.086] | 0.77 |
| LPC 18:0 | 0.59 [0.42, 0.88] | 0.74 [0.46, 1.09] | 0.11 |

The Chi-square test was used for categorical data. Metabolite concentration unit: nmol/mL. Mann Whitney *U* test were used for comparison. LPA 16:0 = lysophosphatidic acid 16:0, LPC-O 18:0 = lysophosphocholine alkyl−18:0, LPC 20:4 = lysophosphocholine 20:4, 12,13-EpOME = 12,13- epoxyoctadecenoic acid, Cer (d18:1_16:0) = Ceramide (d18:1_16:0), TMAO = trimethylamine-N-oxide, HETE = hydroxy-eicosatetraenoic acid, CAR C= acyl carnitines, FA = fatty acids.
